# A Novel Petri Nets-Based Modeling Method for the Interaction between the Sensor and the Geographic Environment in Emerging Sensor Networks

**DOI:** 10.3390/s16101571

**Published:** 2016-09-25

**Authors:** Feng Zhang, Yuetong Xu, Jarong Chou

**Affiliations:** 1 College of Geography and Environment, Shandong Normal University, Jinan 250014, China; zhangfeng.ge@sdnu.edu.cn; 2 Department of Electrical and Computer Engineering, Michigan State University, Michigan, MI 48824, USA; jahhorog.chou@gmail.com

**Keywords:** Emerging Sensor Networks, topology management, Internet of Things, geographic environment, interaction modeling

## Abstract

The service of sensor device in Emerging Sensor Networks (ESNs) is the extension of traditional Web services. Through the sensor network, the service of sensor device can communicate directly with the entity in the geographic environment, and even impact the geographic entity directly. The interaction between the sensor device in ESNs and geographic environment is very complex, and the interaction modeling is a challenging problem. This paper proposed a novel Petri Nets-based modeling method for the interaction between the sensor device and the geographic environment. The feature of the sensor device service in ESNs is more easily affected by the geographic environment than the traditional Web service. Therefore, the response time, the fault-tolerant ability and the resource consumption become important factors in the performance of the whole sensor application system. Thus, this paper classified IoT services as Sensing services and Controlling services according to the interaction between IoT service and geographic entity, and classified GIS services as data services and processing services. Then, this paper designed and analyzed service algebra and Colored Petri Nets model to modeling the geo-feature, IoT service, GIS service and the interaction process between the sensor and the geographic enviroment. At last, the modeling process is discussed by examples.

## 1. Introduction

With the rapid development of embedded computing and new sensor technology, the goal of information and communication technology has developed from connecting anybody to connecting anything at any time and any place. It is hoped that the physical world and the cyber world formed by computing device are connected together more closely, and computing devices can be integrated into people’s lives seamlessly. The more intelligent pervasive services can be provided through sensing the physical world in real time and interacting with the physical world dynamically [1]. Under this background, the Internet of things (IoT) technology comes into being [2]. IoT is an important part of the new generation of information technology, and is the internet where things are connected to things. It extends the internet to the physical world. Through the RFID (radio frequency identification), sensors, and some other technologies, according to the agreement, things in the physical world are connected to the internet for information exchange and communication. Then things can be recognized, located, monitored and managed intelligently.

The IoT can be summarized as three layers: the sense layer, transport layer and application layer. In these three layers there are a large number of heterogeneous devices, transmission protocols, data and so on. These heterogeneities have caused many difficulties of information fusion and sharing. Service Oriented Architecture (SOA) is a component model which can encapsulate the different functional units of applications on heterogeneous platforms to services with well-defined interfaces and standards. These services are integrated together loosely to effectively solve the issues of resource reuse and the interpretability between heterogeneous components [3]. Some researchers have introduced SOA into the IoT. They encapsulate the processes of all devices in the physical world to services, so the IoT can provide its own functionality to the outside world by a unified and universal interface. Interactions can be well done between various heterogeneous devices or between the devices and traditional Web services. The seamless integration of the physical world and the cyber world can be realized.

**Figure 1:**
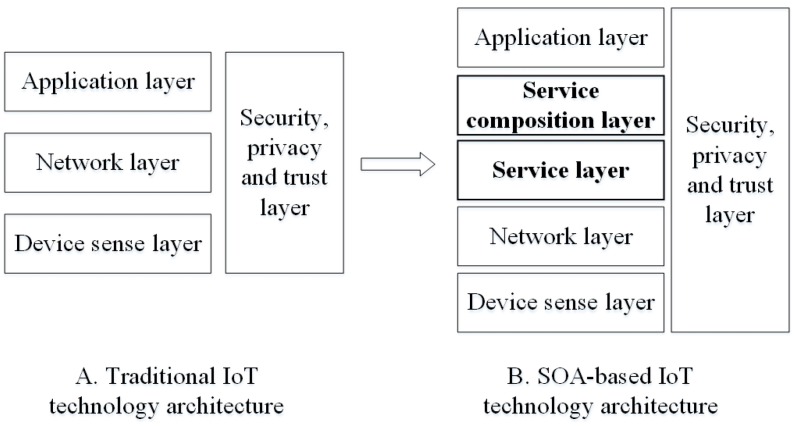
Internet of Things (IoT) technology architecture.

SOA-based IoT technology architecture (Figure 1) introduces the service layer and the service composition layer into the traditional IoT technology architecture between the network layer and application layer [4]. The service layer encapsulates the function of various heterogeneous devices as services, and the service composition layer composes the the device services and traditional Web services to meet the needs of complex application. In the SOA-based IoT technology architecture, services contains two kinds of service: the device service and the traditional Web services. The device service is embedded in a physical device, and can provide real-time data to reflect the state of the physical world. In addition, the device service is always deployed in the resource-constrained device (such as the limited computing, storage capacity, bandwidth and battery).

The existing Web services are mainly oriented to the user and information space. However, the IoT service also should solve the seamless integration and dynamic interaction with the physical space. So the IoT is essentially facing a three-tuple problem domain consisting of user requirement, cyber space and physical space [5]. The IoT service needs to perceive the states of an entity in the physical space in a timely manner, and distribute information to different applications. So the corresponding business system can be triggered to cooperate with each other for achieving business goals.

The sensor devices of the IoT and the sensed entities must be in a certain geographic space. These entities must have geographic locations, and must have certain spatial relationship with each other. If the physical entities which interact with IoT services have obvious geographic spatial distributions, and the information provided by IoT services needs to be processed by GIS (Geographic Information System) technology, we call these IoT applications “GIS-oriented IoT service applications”. Because of the unique information integration ability, the visualization and the powerful spatial analysis ability, GIS has been widely used in the industry and has played an important role as an important tool, technique and discipline for collecting, processing, managing and analyzing the geographic spatial data. Many IoT applications are inseparable from GIS technology. For the IoT, GIS could provide the key technical support, enrich the means of information storage and management, enhance the data analysis and mining capabilities, strengthen the level of information management and make the combination of IoT and GIS more and more closely.

The combination of IoT and GIS has many application areas, such as urban planning, disaster warning, logistics monitoring, municipal management, etc. In these applications, IoT services provide the information which is sensed from geo-entities. GIS analyzes and processes these information, and then provides decision support to the related personnel or processes the relevant business automatically. However, the functionality of IoT sensor is relatively simple in general, and the sensed information is very limited. The single information from one kind of sensor is not enough for most of the integrated application of GIS. Many times the state of a geographic entity (geo-entity) needs to be described by the information from various of sensors.

For example, a landslide prone slope monitoring needs at least four types of sensors, including the rock fracture deformation monitoring device, tension monitoring device of collapse prevention network, rock body tilt monitoring device, automatic rainfall monitoring device. The information sensed by all these sensors could describes the state of this slope. Only one type of device is not enough. In another example, the early warning of fire needs sensors of humidity, temperature, CO, CO_2_, wind speed, wind direction and some other information. A kind of sensor cannot meet the needs of fire warning. Therefore, only the information combined with multiple types of sensed information could provide more powerful decision support for the application. All these information is provided by the IoT services, and these various information describes the state of a geo-entity (such as a slop, a fire monitoring point). So, how to describe and model these IoT services and the corresponding geo-entity, and how to put the information provided by these IoT services into the GIS applications become problems worthy of study.

The spatial analysis of GIS is very powerful. From the object-oriented perspective, the basic object of spatial analysis is the geographical feature (geo-feature). In GIS, geo-feature is the abstraction of real world phenomena [6], and can be regarded as the mapping of a geo-entity from real world to information world. In the process of business modeling and applications, if we only consider the information from all kinds of IoT services without considering the corresponding geo-entity, then we would only see the information of scattered numbers or strings, and it is not easy to see the relationship between all kinds of information. The relations of these device services are fuzzy too. These fuzzy relations would result in confusions of understanding and application, and cause great inconvenience for the subsequent spatial analysis. Therefore, in GIS-oriented IoT services applications, we combine all the sensed information about one geo-entity, and attach this information to the geo-feature which is mapped by this geo-entity. We can use this geo-feature to analyze the temporal and spatial distribution of all kinds of sensed information through the spatial analysis of GIS efficiently. Then we would achieve the goals of business applications. For example, in the research of distribution of geological disaster prone areas, if we map the slopes to geo-features, and map all kinds of sensed information provided by IoT services to the characteristics of the geo-features. All the sensed information and the corresponding slope would be combined to a spatial analysis object. Then we could analyze the spatial distribution or the spatial relationship of geo-features through the GIS spatial analysis method. All the sensed information is no longer discrete, but a tight whole. This idea can greatly improve the understandability of business modeling and application, improve the efficiency of business analysis, and also become more suitable for spatial analysis of GIS.

For GIS-oriented IoT service application, this paper discusses the IoT service modeling method from the perspective of geographical space, and try to combine the IoT service, the GIS service and their common descripted object (the geo-entity) together to analyze and model the geo-entity, the IoT service, the GIS service and their relationship. This paper combines the information provided by multi IoT services and the corresponding geo-entity to a geo-feature in the information space. By this way, it is not discrete information but geo-feature that flows between IoT services and GIS services. So the modeling process and business model are easier to understand, and are more suitable for spatial analysis.

This paper regards the modeling process of GIS-oriented IoT service application as a service composition process, and models the composition of IoT service and GIS service using the service algebra and Colored Petri Nets (CPN). This model ensures the reusability of the composited services in a large extent, and ensures the analyzability and scalability.

The remainder of this paper is organized as follows. Section 2 introduces the related work of IoT service modeling and service composition. Section 3 discusses the basic elements and formal expression of GIS-oriented IoT service application. Section 4 discusses the service algebra, CPN and the modeling process of GIS-oriented IoT service application. Section 5 uses an example to illustrate and discuss the modeling process. Section 6 is a summary of the whole paper, and points out the direction of our ongoing work.

## 2. Related Work

The research work related to this paper includes two aspects: IoT service modeling and Web service composition.

### 2.1. IoT Service Modeling

With the development of IoT, many researchers begin to solve the heterogeneous problem of IoT devices and applications using the service oriented approach, but their research perspectives are different.

Due to the reasons of deployment environment and cost, most embedded devices of IoT are constrained by resources, such as the limited storage, the limited power supply, etc. [7]. Many researchers are studying the resource-limited devices of IoT. For example, the wireless sensor nodes must be capable of being active for a long time without battery recharge. This increasing necessity asks for technologies and methods to anticipate the level of energy drain in these devices. The paper [8] proposed a modelling approach based on Fluid Stochastic Petri Nets to estimate single node performance in the presence of several energy consuming entities and obtained good simulation results.

So, according to the limited resources, the IoT service needs some service standards which are as concise as possible. However, traditional Web service standards, such as WSDL and SOAP, do not consider the resource constrained conditions, so they are not suitable for the IoT service. Therefore, some researchers study the IoT service from the perspective of resource constrained characteristic, and explore how to achieve more functionalities in the case of less occupied resources. The paper [9] defined a lightweight service protocol stack DPWS (Devices Profile for Web Service). Under the premise of supporting secure messaging, service description and discovery, DPWS provides a concise subset for Web services standards of physical equipment. It means that the service provided by the equipment can be seamlessly integrated into enterprise applications, and it is now widely used in IoT.

Based on the idea of SOA, the paper [10] proposed SODA (Service Oriented Device Architecture) to provide a high level abstraction for the physical world device. Based on service SODA constructed a bridge between physical world and information world, so that developers can connect a variety of IoT services to enterprise service bus and the user can access the IoT service like accessing the Web service. The paper [11,12,13] used DPWS to describe the service of the equipment level, and proposed a Web service-based shop floor integration infrastructure which is called SOCRADES. Thus based on Web service technology SOCRADES linked closely the intelligent manufacturing equipment in the workshop with the high level background system such as ERP system. Based on Petri Nets, the paper [14] proposed a feasible lightweight model EPN and a description language EPNML to describe the behavior of IoT service and business process including process control and logic relationship structure. This model increases the effectiveness and scalability of IoT services.

In order to improve the description ability of IoT, some researchers have studied the modeling, selection and verification of IoT service from the perspective of service interaction. The paper [15,16] put forward the concept of Service Gateway which is regarded as the mediator between the internet and embedded system. It can explain and convert the transmitted information of both sides, so that they could be combined seamlessly. However, both of them retain their respective description language and communication protocol. From a perspective of Web service interactive environment, the paper [17] used timed automata and TCTL (time computation tree logic) to express the time-related behaviors and properties of the IoT service respectively, and used the model checking tool UPPAAL to verify the timeliness and correctness of the IoT service. The paper [18] used Markov decision process and PCTL (probabilistic computation tree logic) to describe the reliability and the resource consumption-related behaviors and properties of the IoT service, and used the probabilistic model checking tool (PRISM) to verify them. Then the strategy of the IoT service composition which is suitable for the certain reliability and resource consumption could be selected. The paper [19] proposed an IoT service behavior modeling method. This method models the IoT service and physical environment as probabilistic timed automata, and describes the user expected service characteristics as temporal logic formula. For the IoT service, this method could verify the correctness of the functional behaviors and the satisfiability of nonfunctional constraints. The hybrid system has the depicting ability for the discrete command of information system and continuous behavior of physical equipment. So based on hybrid system theory, the paper [20] put forward an IoT service modeling and verification framework. In this framework the hybrid system is constituted by the physical equipment and the corresponding control system, and the atomic service is constituted by the hybrid systems. The composed services are composed by the atomic services which are organized by the distributed hybrid systems. This framework effectively unified physical device, control system and composed services system, and have the deep modeling and comprehensive verification ability for the IoT service.

In order to improve the accuracy and efficiency of the service discovery, some researchers have studied the IoT services modeling from the semantic perspective. The paper [21] discussed the semantic integration of service, and added the related domain knowledge to the description of various services. So the service discovery becomes more efficient and accurate. The paper [22] proposed a semantic modeling method for IoT service. This method discussed a group of core concepts (equipment, entity, resource and service) and their relationships in the IoT semantic model. It uses OWL-DL to describe entities and resources, and uses OWL-S to represent the IoT service. This method associates services, entities and resources closely together, so that it can provide a unified interface description for all kinds of objects in the IoT. Through semantic information tagging the machine can understand the description of the service. The accuracy of the automatic service discovery is improved.

In addition, there are researchers using the perspective of user and context for the IoT service model. The paper [23] proposed an IoT-based user-driven service modeling environment. This modeling environment makes it easy to create IoT services for the non-technical users, and also provides the process of generation and description of the context information based on ontology. According to the dynamic availability and context of the IoT, the paper [24] presented an environment-based approach for modeling the IoT services. Based on WSMO-Lite, this approach provides an environment entity model for capturing context information. The paper [25] proposed a user-centric IoT-Based Service Framework SoIoT (Service-Oriented Internet of Things) which can integrate the IoT service in the urban computing environment. This framework provides a task-oriented algorithm which can promote the spontaneous composition of the IoT service.

The GIS-oriented IoT service application has its particularity, i.e., spatial characteristics. The geo-entity which is the object of description and interaction of the IoT service not only has the geographical location, but also has complex spatial relationship. This paper discusses the behavior characteristics of the IoT service, the geo-entity and the business-related GIS service. Then this paper analyzes their relationship from the perspective of information flow. According to these discusses and analysis this paper models the GIS-oriented IoT service application.

### 2.2. Web Service Composition

Web services are independent and modular software applications, and can provide unique functions or interoperability between different existing applications [26]. However, an IoT service only has a single function, and can only sense one characteristic of a geo-entity in general. In most cases a single IoT service is unable to meet the business goal. Web service composition is a promising solution which can compose the functions of two or more Web services into one service to meet the needs of user [27]. Therefore, it is necessary to compose multi IoT services and other related services in a certain way to meet the requirements of complex business applications. At present, there are many researchers studying the service composition. The theories or technologies consist of the workflow technology, the artificial intelligence method, the formal method, etc.

The workflow-based composition method usually achieves the business goals in a semi-automatic way. The workflow system provides the graphical interface of the service composition. The service composition designer defines the abstract service flow through the man-machine interaction, and the service matching and binding are processed by the system automatically. This method makes full use of the domain knowledge of the designer, and reduces the complexity of the system and the workload of the designer. It is easy to realize, and has better dynamic and flexibility.

Based on workflow, the papers [28,29,30] provided service templates for service composition. Through service templates, the designer can set the service function, input, output and QoS semantic information. So the designer can describe the target service, define the combination process and generate the combination scheme.

The paper [31] presented a semantic Web service composition method based on Workflow ontology. Through constructing the ontology relationship which could express the relationship between business process and services, this method uses the hierarchical relationship of service in ontology and the similarity between semantic concepts to realize semantic Web services composition automatically under the support of formal reasoning technology.

By establishing the domain task ontology the paper [32] proposed a service composition method which can generate the workflow dynamically. The paper [33] proposed a workflow automatic generation system. This workflow system can find a suitable service set, and in this set the appropriate services are chosen to compose a workflow for the user’s functional requirements and nonfunctional requirements. The system needs to build the domain knowledge base, workflow and Web services, and then use an algorithm to generate the workflow automatically according to the request of the Web service requester. It reduces the semantic fuzziness in the search process through the classification of Web services and workflow. In order to save battery life, improve storage capacity, the paper [34] used Windows workflow to compose Web services for cloud based mobile application.

The method based on artificial intelligence has a flexible description about the dynamic evolution system, so it is more suitable for describing the dynamic behavior of the service composition. Artificial intelligence technology includes intelligent planning, situation calculus, etc. For the early intelligent planning, people used HTN to compose services mainly. The paper [35] developed SHOP2 (Simple Hierarchical Ordered Planner2) and used the HTN (hierarchy task network) technology successfully in Web service composition. SHOP2 decomposes a task into very small original tasks which can be executed directly in accordance with the planned order of SHOP2. Based on this, the paper [36,37] expanded and developed this method to improve its practicability.

Based on DAML-S and Agent the paper [38] proposed an automatic service composition method through expanding the situation calculus-based logic programming language Golog. First, this method uses OWL-S to describe the semantic of Web service from the level of operation, data and service. Then this method uses the precondition of activity in situation calculus to describe the precondition of every atomic process, and converts the Web service semantic description model to the axioms of situation calculus. Thus the service composition problem is transformed to a program execution problem that meets the target attribute.

The paper [39] proposed a multilayered, rule-based combination model. It uses predefined rules to calculate the semantic compatibility between different services, and uses the specification language of combination request to describe the combination demand. So it can use the rule and service template to composite services in semantic web, and it has been successfully applied to the field of e-government. The paper [40] proposed a rule-based service composition method with minimum execution cost. This method uses rules to model the Web service, and uses the parameter ontology to eliminate the semantic conflict. Meanwhile this method defined a deductive web and uses backward deductive method to discover service, compose service, and produce the optimal composition scheme.

Formal method has strict mathematical reasoning and proof, can describe the service composition process dynamically, and can verify the correctness and validity of service composition. So many researchers study the service composition by formal methods. Formal methods consist of Petri Nets theory, process algebra, description logic, etc.

The paper [41,42,43,44] used the *π*-calculus to describe and model Web services and their composition formally, and used formal tool to verify the correctness of the service composition. The paper [45] also used *π*-calculus to verify the demand satisfaction of service composition.

Based on the traditional description logic, the paper [46] constructed the dynamic description logic to describe the dynamic behavior of Web service, based on which an agent-based semantic Web service composition is proposed. This method converts the semantic Web service to the action of formal intelligent agent. Then this method is able to model semantic Web services under the support of knowledge base. It uses the target-driven, autonomous and reasoning characteristics of agent to provide effective support for the automatic discovery and composition of semantic Web services. The paper [47] regarded each input or output of the Web service request as the concept of the description logic and presented a service-based dynamic composition algorithm according to the semantic similarity of the given five kinds of description logics.

The paper [48] defined a Petri Nets model to describe the Web service and analyzed service simulation, verification, and automatic composition. This automatic combination has several kinds of basic mode such as sequential, parallel and etc. At first this model uses the available Web service to produce all possible service combination Nets according to their interaction, and then chooses the suitable composition which can reach the target marks in these composition Nets. The complexity of this method depends on Petri Nets and all possible service compositions.

The paper [49] defined the behavior of Web service as Petri Nets and introduced the corresponding Petri Nets representation of the sequence, condition, iteration and parallel operation. The paper [50] defined the rules to transform BPEL4WS into a service-oriented Petri Nets (BPWF-Net) which is used to analyze the correctness of service composition.

The paper [51] established a service oriented architecture WS-net to describe Web service. WS-net uses Petri Nets to define three levels including interface, communication and interoperability. Each level consists of two channels: “Enqueue” which receives data and “Dequeue” which receives and processes the service request. This model is good for the reliability verification of software services and could use Petri Nets theory to analyze the system.

The paper [52] converted Web services to a set of horn clause rules, and converts the input and output of user request to the fact and goal in the horn clause. Thus the satisfiability problems of user request are converted to the logic reasoning problems in horn clause. In this paper, Petri Nets is used to model horn clause, and T-invariants technology is used to determine the satisfiability of user request.

In addition, there are some other methods proposed by researchers for service composition. The paper [53] used graph theory to model the service composition. A service composition map is formed by composite operations, and the relations between operations, invokers or requests can be regarded as a composition graph.

Some researchers are studying the evaluation of service composition. Many complex SOA applications are based on services composition and the non-functional properties evaluation is very important. For performance evaluation of SOA-based applications integrated by BPEL the paper [54] presented an approach based on a performance-oriented reinterpretation of the BPEL specification as a performance modeling language within a multiformalism framework. Based on automatic translation of PerfBPEL into Markov chains, this approach is implemented by means of SIMTHESys modeling and analysis framework to enable the interaction with other performance oriented formalisms.

The GIS-oriented IoT service composition is the composition of the IoT service and the GIS service. Compared with the general Web service, the IoT service and the GIS service have their own characteristics. The IoT service is affected by the environment seriously and is resource-constrained. The GIS service has characteristics of large data quantity, complex data analysis, significant multi-dimension, complex spatial relations and so on. Meanwhile, the IoT service and the GIS service all have close relationship with the geo-entity in real world. Therefore, it is needed to design a more suitable service composition method for GIS-oriented IoT service applications. Each kind of existing service composition methods have advantages and disadvantages and are in constant development and improvement. It is difficult to judge which one is best, and we can only judge which one is suitable for a specific application scenario. Petri Nets has both strictly mathematical definition and graphical description and could verify the effectiveness and feasibility of service composition more easily. In our research, from the perspective of modeling and analysis, Petri Nets is used as a service composition method to model GIS-oriented IoT service application for deep analysis and validation of the model in the whole research.

## 3. GIS-Oriented IoT Service

GIS is a computer system whose task is to collect, store, manage, retrieve, analyze and describe the distribution of spatial entities and related attribution data and to answer questions raised by users in the support of computer hardware and software [55]. The IoT must exist in a certain geographical space, and the sensor must have relations with the geo-entity in geographical space. If the entity which interacts with the IoT service have obvious geographic distribution characteristics, and the information provided by the IoT service needs to be processed by GIS technology, we call these IoT service applications “GIS-oriented IoT service applications”.

In GIS-oriented IoT service application, the geo-entity in geographical space is the sensed object. The IoT sensor device senses information from the geo-entity and shares the information by IoT service. GIS service obtains the information, and processes and analyzes the information in accordance with the business rules to achieve business goals. If it is necessary, IoT service would adjust the state of related geo-entity. Therefore, in GIS-oriented IoT service application, the basic three components are the geo-entity, the IoT service and the GIS service. The modeling method discussed in this paper mainly considers these three parts.

In this section we first introduce Petri Nets which is used for modeling, and then we will respectively discuss the geo-entity, the IoT service, the GIS service and their formal representation. Finally, we will discuss their close relationship from the point of view of information flow.

### 3.1. Petri Nets Introduction

Petri Nets [45] is composed of Place which represents state and Transition which represents changes. It is suitable for describing asynchronous concurrent system. When the places and transitions in the Petri Nets model are correctly described, the Petri Nets theory could verify the correctness, reliability and some other properties of this model. Therefore, Petri Nets is suitable for loosely coupled Web services and can strictly control their behaviors.

Colored Petri Nets (CPN) [56] is a kind of High-Level Petri Nets which classifies places for Net system folding. Because there is no concept of data in Petri Nets, all the data control must be converted to net structure, which leads to a very large model. CPN combines advantages of Petri Nets and high-level programming language, and can be used to simulate complex system with graphical concept. CPN has capabilities of dynamic simulation and formal reasoning for system behavior. Based on Petri Nets CPN introduces the concept of data type and data operation to reduce the basic elements of Petri Nets. Thereby CPN reduces the size of the net system, enhances expression ability and makes the model clearer and easier to understand. For more information, please refer to the relevant literature about CPN and Petri Nets.

**Definition** **1.** *Colored Petri Nets (CPN) is a 8 tuple CPN = (Σ,P,T,F,C,G,E,I) where:*

(1) Σ *is a finite set of types, called color set.*
(2) P is a finite set of places.
(3) T is a finite set of transitions.
(4) F is a finite set of arcs such that: F⊆P×T∪T×P;P∩T=F∩P=P∩F=∅.
(5) C is a color function. C:P→Σ.
(6) G is a guard function, G:T→B.∀t∈T:[Type(G(T))=B∧Type(Var(G(t)))⊆Σ].B∈{false,true}.
(7) E is an arc expression function, $E:F\to \;B;\;\forall \;f\;\in \;F:\;[Type\left(E\left(f\right)\right)=C{\left(p\left(f\right)\right)}_{M_{S}}\wedge \;Type\left(Var\left(E\left(f\right)\right)\right)\subseteq \Sigma ]$, where p is the places connected to f.
(8) I is an initialization function. I:P→Σ creates the start mark $M_{S}$ for the color of every places, i.e., $\forall p\in P:[Type\left(I\left(p\right)\right)=C{\left(p\right)}_{M_{S}}]$.

### 3.2. Geographic Entity

The physical entity in the IoT generally refers to the sensed object, sensor, and information processing facility [57]. The geo-entity (geographic entity) studied in this paper is a part of the sensed object and is a subset of the physical entity. Geo-entity has obvious characteristics of geographical spatial distribution and has complex spatial relationship in geographical space.

The requirements of GIS-oriented IoT service application are completed through the interaction between the device and the geo-entity. The basic element of geographical space is the geo-entity which can be defined as a phenomenon that occupies a position in geographical space. Geo-entity cannot be divided into similar phenomena in the real world and can be expressed as simple abstract geometric objects including the point entity, the line entity, the surface entity and the body entity [58]. A city, a car moving on the road or an air quality monitoring point all can be regarded as a geo-entity.

Geo-entities in the real world have a complex spatial relationship with each other. The distribution relationship of geo-entity in the geographic space is called spatial relationship including topology, direction and measure relationship. The topological relationship consists of adjacent, correlative and containing relationship. The direction relationship consists of the position relationship. The measure relationship mainly means the distance relationship.

The geo-entity has three characteristics including spatial, attribute and temporal characteristics. Spatial characteristics which are also called the geometric or topological characteristics describe the location and spatial relationship between geo-entities. Attribute characteristics describe the attributes of geo-entities. Temporal characteristics describe the presentalism of geo-entities.

The geo-entity is the basic component of real world, is the sensed object of IoT and is the basic part of GIS-oriented IoT service application. For example, a self-driving car can be regarded as a geo-entity inside which there are a large number of sensors. These sensors common sense some specific properties of the car, and control the car according to the sensed information. We take the location information as an example. The GPS devices can sense the location information of the car. The interaction between the car and the location sensor consists of three parts: (1) the sensor senses the location information of the car; (2) according to the location information and the surrounding road information, spatial analysis gives the correct driving guide; (3) the corresponding sensor adjust the speed and direction of the car according to the guide. So according to the relationship between the geo-entity and the IoT sensor, the geo-entity can be classified into two categories:

- **Sensed geo-entity:** The attributes of the sensed geo-entity can be sensed by sensors. For example, the direction, velocity and location of a self-driving car can be sensed by sensors; the pollution indicator of a lake can be sensed by the relevant sensor. At the same time, the geo-entity also has its own evolution behaviors of the state. For example, the pollution indicator of a lake change with time. The evolution behavior of the lake, such as the chemical reaction and the biological process, cause the attribute value(the pollution indicator) of the lake to change constantly. So it is needed to sense the geo-entity by the IoT sensing device constantly.
- **Controlled geo-entity:** For this type of geo-entity, not only its attributes can be sensed, but also its attributes can be changed by commands received from the sensor. So compared to the sensed geo-entity we need to have a better understanding of the behavior of the controlled geo-entity. The different attribute values of this kind of geo-entity are correspond to its different states. So the sensor could change the state of a geo-entity through changing its attribute values.

From the object-oriented perspective, in GIS the basic object of spatial analysis is the geo-feature which is the abstraction of real world phenomena. Through GIS, the geo-entity in geographical space can be mapped to the geo-feature in the information space, that is, the geo-feature is the expression of the geo-entity in the information space. Based on the above analysis, the following gives the formal expression of geo-feature.

**Definition** **2.** ***Geographic Feature:**GF is a geo-feature which is the mapping of a geo-entity in the information space. It can be sensed or controlled by the IoT sensor service. The geo-feature can be represented as a 7 tuple GF=(GFID,SC,AC,TC,BF,OP,SD) where:*

(1) GFID is the unique identifier of GF.
(2) SC denotes the spatial characteristic set of GF, including location coordinate set, entity type, area, perimeter, spatial relationship with the other geo-features and so on. Location coordinate set can be obtained from IoT service. Area and perimeter can be obtained through the related GIS service. The spatial relationship such as inclusion, intersection can be obtained by the spatial analysis of GIS service.
(3) AC denotes attribute characteristic set of GF, and TC denotes temporal characteristic set. They all can be obtained from the IoT service.
(4) BF denotes the behavior and function of GF. BF can be inherited from the type of this geo-feature. For example, the geo-feature of a weather monitoring point can inherit the behavior and function from the point type feature of GIS. BF also can be defined according to the specific scenario.
(5) OP is the allowed operation set of GF. ∀op∈OP,op=(Operation,Content). Operation is the operation behavior, Content is the operation content. For example, op=(Switch,on/off) denotes that the allowed operation of the geo-entity corresponding to GF is to switch on or to switch off. OP is defined according to the specific scenario.
(6) SD is the semantic description of GF.

### 3.3. IoT Service

IoT extends the internet to a variety of information sensors such as RFID, wireless sensor, global positioning system and so on. These sensors can provide all kinds of basic services which are called IoT services. The basic element of geographical space is geo-entity. The geo-entity reflects the state of geographical space, and the IoT service maps the sensed information of the geo-entity to the information sapce. The interaction between them reflects the integration of geographic space and information space. All geo-entities are independent of the IoT services and they are not attached to any one of the IoT services.

The ability of the IoT service is reflected in the interaction with the geo-entity. The interaction consists of sensing and controlling the geo-entity. On the one hand, the IoT service can provide the sensed attributes information of geo-entity, such as the temperature of a location, the water pollution indicators of a lake, etc. On the other hand, the IoT service can change the state of a geo-entity according to the sensed information in a direct or indirect way. For example, the IoT services in a self-driving car can adjust the direction and speed of the car according to the sensed location or speed information.

The IoT application system is a kind of information system for some kind of business application. It must be able to meet the expectations of user and realize the business logic. IoT service is a kind of software service in this information system, which can implement the specific business logic. The business logic is reflected by IoT services to the sensed or controlled geo-entity. The IoT device services can be classified into two categories [19] according to its function and the interaction between the sensor and the geo-entity.

- **Sensing Service.** Through interaction with the geo-entity, this kind of service collects the information, senses the state (attribute values) changing of a geo-entity and shares the sensed information to other services or applications. In general, the sensing service periodically collects the information of the sensed geo-entity, but one sensing service only has the ability to sense one property of one geo-entity. For example, the GPS sensor inside the mobile phone can only sense the location of the phone, and a temperature sensor in a location can only sense the temperature information of this location.
- **Controlling Service.** This kind of service first receives the control information from other services, and then changes the attribute values (state) of the geo-entity directly or indirectly. The direct way is to convert the control information into the control commands which the controllable geo-entity can accept, and then send it to the corresponding controllable geo-entity to adjust its state directly. For example, if a self-driving car senses the route deviation, then the related sensor device would adjust it direction or speed directly. The indirect way is not to change the attribute values of a geo-entity directly, but take other ways to change the attribute values. For example, if the lake water pollution reaches to a certain degree, the related sensor devices would release some biologic to alter the water pollution indirectly.

According to the difference between the IoT service and the general Web service, it can be concluded that the GIS-oriented IoT service has the following characteristics:

- **Real Time.** The sensor must sense the state of a geo-entity in real time, and provide real-time IoT service. According to the sensed information the controlling IoT service must control the controlled geo-entity in real time. The sensed information and control demands of IoT services must be in real time. For example, a sensor monitors the carbon dioxide concentration in a region, or a GPS sensor tracks the location of a vehicle. All these scenarios have strict requirements for real time.
- **Temporal and Spatial Distribution.** The sensed or controlled object of GIS-oriented IoT are the geo-entity in geographical space, and the most obvious characteristics of geo-entity are its temporal and spatial distribution characteristics. The spatial distribution characteristic is characterized by its geographical location attribute, and it has a certain spatial relationship. The temporal distribution characteristic is characterized by its state changing with time. As a result, the information described by GIS-oriented IoT service has temporal and spatial distribution characteristics, and the behaviors of the IoT service need to be adjusted in time with the change of the geo-entity. For example, for a continuous moving self-driving car, its location is constantly changing, and its direction should be adjusted according to the changing location information. Here the location is a temporal and spatial distribution characteristic of the car. What the general Web service describes and interacts with is the business information in a business application. It is a kind of virtual entity, and generally does not have the temporal and spatial distribution characteristics.
- **Strong Interactivity.** The GIS-oriented IoT service interacts with the geo-entity in the environment through the IoT device. The IoT service senses the attribute of the geo-entity by the sensing device, thus it can monitor the state of the geo-entity in real time. IoT service share the sensed information (the state information of the geographical entity, such as the location of a car and so on) to GIS service for business analysis ( for example, the GIS spatial analysis service analyzes the spatial relationship between the car and the road to judge whether the route is correct), and adjust the state of the geo-entity by the controlling service to meet user expectations.
- **Dynamic Service Quality.** The device which provides the IoT service is often deployed in the complex geographical environment and uses the wireless self-organized way to interconnect. So there are many reasons that cause the IoT services to be dynamic and unstable. For example, because of the natural environment (such as the rainfall, wildlife damage, etc.), energy shortage of equipment or constrained resource [59], the service quality would decline or even disappear. However, the business applications require the IoT services to provide a reliable service.

The physical device which provides the IoT service is deployed in the natural environment, and is often resource constrained, such as storage capacity, computing power, electricity and so on. So the device service in general is atomic service [20]. Atomic services are ones which cannot be decomposed into finer grained services, and are ones where a single web-accessible computer program, sensor, or device is invoked by a request message, performs its task and perhaps produces a single response to the requester [60]. The following are formal descriptions of IoT services.

**Definition** **3.** **Sensing Service:** Sensing service provides the sensed information of a geo-entity. Due to constrained capacity, the sensing service involves only one geo-entity and is corresponding to one attribute of the geo-entity. If there are multi sensing services providing different attributes of the same geo-entity, then these services can describe the state of this geo-entity. The formal representation of sensing service is a 7 tuple: SIoTS=(SSID,URL,GFID,GSA,GSV,SD,STT), where,

SSID is the unique identifier of sensing service. URL is the invocation of this service. GFID is the unique identifier of the geo-entity sensed by SIoTS. GSA is the type of sensed information. GSV is the value of sensed information. GSA and GSV are corresponding to the characteristics of the geo-feature. SD is the semantic description of SIoTS. STT is the description of state of SIoTS, such as available,unavailable.

**Definition** **4.** **Controlling Service:** According to the business goals, the controlling service will control and adjust the state of the related geo-entity after analyzing the sensed information provided by the sensing service. The controlled object of this type of service is the controllable geo-entity. The formal definition is a 7 truple CIoTS=(CSID,URL,GFID,GFCT,GFCC,SD,STT) where:

CSID is the unique identifier of controlling service. URL is the invocation of this service. GFID is the unique identifier of the geo-entity controlled by CIoTS. GFCT is the type of control action. GFCC is the command of control. GFCT and GFCC are corresponding to OP=(Operation,Content) in Definition 2. GFCT is corresponding to Operation, and GFCC is corresponding to Content. SD is the semantic description of CIoTS. STT is the description of state of CIoTS, such as available,unavailable.

### 3.4. GIS Service

**Figure 2:**
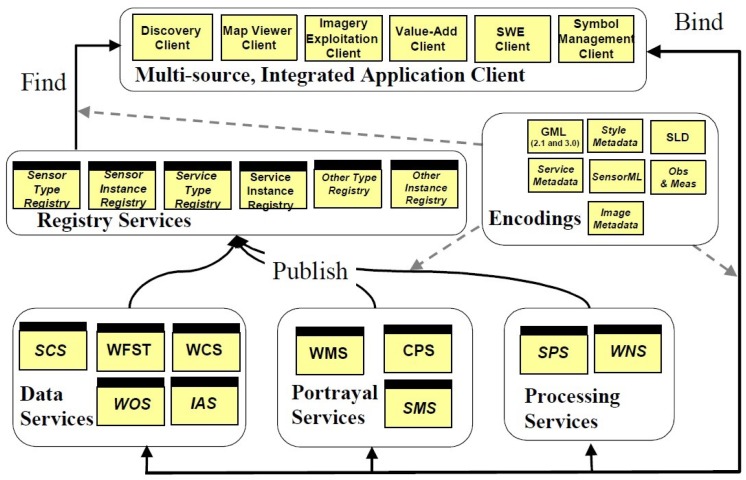
OWS framework [61].

OGC (open geospatial consortium) is an international not for profit organization committed to making quality open standards for the global geospatial community. In order to extend the concept of GIS service to the internet better, OGC established OWS (OGC Web service) research program since 1999. OWS service development follows the OWS service framework which defines a standardized service, interface, and exchange protocols. These standards are applicable to applications. Figure 2 [61] shows OWS framework. It can be seen from the figure that the services in OWS can be classified into five categories including Data services, Portrayal services, Processing services, Registry services and Application services. Data services, Portrayal services and Processing services are published in the Registry services. Application services find services through the Registry services, and then bind and execute services according to the query results. In these service categories, the services related to this paper are Data services and Processing services.

#### 3.4.1. GIS Data Service

Data services provide the access to data sets stored in a data warehouse and database. WMS (Web Mapping Service), WFS (Web Feature Service) and WCS (Web Coverage Service) of OGC are all Data services. The Processing services can analyze the data from these data services using various types of spatial analysis methods.

**Definition** **5.** ***GIS Data Service:** A GIS Data service can be described by a 7 tuple GISDS=(DSID,URL,QoS,DST,GFT,ATT,SD) where:*

(1) DSID is the unique identifier of GISDS.
(2) URL is the invocation of the GIS Data service.
(3) QoS is the description of service of quality. Sometimes we should select the suitable service from several services, the QoS is a good reference indicator. QoS=(cost,time,reliability,reputation,integrality,accuracy). The first four including cost,time,reliability,reputation are consistent with the QoS description of the general Web service [62]. The latter two including integrality,accuracy are special description for geospatial data service. The spatial data provided by GIS Data services could describe the realistic geographical phenomenon, and integrality denotes the data integrity which is the completeness degree of this type of description. Any information of geo-entity should not be missed out. Accuracy denotes data accuracy which is the proximity between the spatial data provided by GIS Data service and the realistic geographical phenomenon. Due to the spatial, attribute, temporal characteristics are three basic elements for the expression of spatial information, the accuracy of the data can be measured by positionaccuracy$A_{s}$ (the proximity between the coordinate data and the real position), attributeaccuracy$A_{a}$ (the consistency of attribute value and the real value) and temporalaccuracy$A_{t}$ (the presentalism of the spatial data).
(4) DST is the type of Data service, DST∈{WMS,WFS,WCS,WOS,...}. If DST=WFS, then this data service can provide geo-feature set which contains the same type of geo-features in a certain geographical region. The type of geo-feature set includes point type, line type, polygon type in general.
(5) GFT is the type of the geographic feature, such as point, line, polygon and so on.
(6) ATT is the attribute characteristic set of geo-feature. Some attribute characteristics are corresponding to the information provided by sensing service.
(7) SD is the semantic description.

#### 3.4.2. GIS Processing Service

**Figure 3:**
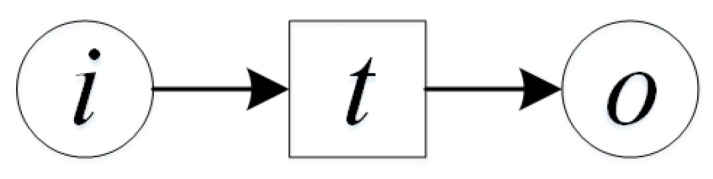
CPN model of PS.

GIS processing service is the basic application service which is able to perform some operations on the spatial data and provide value-added functionality, such as spatial query, buffer analysis, overlay analysis, etc. The processing service usually has one or more input and produces the corresponding output after value-added operations. Processing service is able to convert, merge or create data, and is able to establish a loose or close association model with data services. So for such service, the input mainly is geo-feature, the output is the expectation of user, which is the decision supporting information or control command for some geo-entities (Figure 3).

**Definition** **6.** ***GIS Processing Service:** GIS Processing Service can be described by a 5 tuple PS=(PSID,URL,QoS,SD,PSCPN) where:*

(1) PSID is the unique identifier of this processing service.
(2) URL is the invocation of the GIS processing service.
(3) QoS is the description of the quality of service.
(4) SD is the semantic description.
(5) *PSCPN=(Σ,P,T,F,C,G,E,I) where:*
  - Σ *is a color set, denotes the input and output geo-feature type and other information type. The definition of colors includes:*
    Color GFT: the geo-feature,
    Color STR: the other information, such as number, string.
  - P is a finite place set, $P=\left\{i,o\right\}.^{•}i=\emptyset ,o^{•}=\emptyset .$ i is the input of processing service, i.e., geo-features or some other information. o is the output of processing service, Type(i),Type(o)∈{GFT,STR}.
  - T is a finite transition set, denotes the internal operations of the processing service, that is, the process of analyzing the input geo-features and other information and outputting the results. For example, the processing service of buffer query needs the input of the target geo-feature and the buffer distance, and outputs the polygon feature with position, area, perimeter and some other attributes after the buffer analysis.
  - F is a finite arc set. F={(i,T),(T,o)}.
  - E is the arc function set. E={E(i,T),E(T,o)}.
  - C is the color function. C={C(i)=GFT∪STR,C(o)=GFT∪STR}.
  - G is the guard function which is defined according to the specific service. G can determine whether the processing service could be executed.
  - I is the initialization function which can confirm the initial conditions, I:P→Σ is the initial mark $M_{S}$ produced by the color value of every place. $\forall p\in P:Type\left(I\left(p\right)\right)=C{\left(p\right)}_{M_{S}}\wedge p\notin i\to I\left(p\right)=\;\emptyset .$

#### 3.4.3. Information Flow in GIS-Oriented IoT Service Application

**Figure 4:**
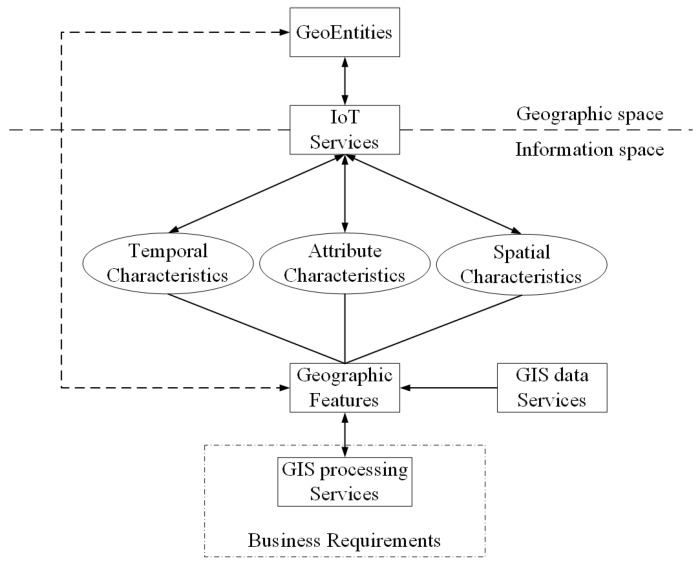
The information flow of GIS -oriented IoT service application.

Figure 4 describes the flow of information among geo-entities, IoT services, GIS services, and business activities. In this figure there are two spaces including geographical space and information space. Geographical entity belongs to the geographical space, the IoT service is a bridge between the information space and the geographical space, and it can reflect the geographical entity to the information space. The geo-feature and GIS service all belong to the information space. On the one hand information flows from the geographical space to the information space, and then returns from the information space to the geographical space after the business analysis. The following will discuss the information flow in these two spaces according to the flow direction.

(1) From the geographical space to the information space:

- The IoT senses the geo-entity by the sensor and uses the sensing service to share the sensed state information of the geo-entity, such as spatial location, temperature and other information.
- The information from sensing services and related GIS data service (if needed) are integrated together to constitute a geo-feature. Then the geo-entity is mapped to geo-feature of information space, and the information provided by sensing services is mapped to the spatial characteristics, attribute characteristics or temporal characteristics of this geo-feature.
- The GIS processing service takes the geo-feature as the basic object of spatial analysis to process business requirements.

(2) From the information space to the geographical space:

After spatial analysis, if the business requires to adjust the state of some geo-entities, then there will be a reverse process. The information returns back.

- After spatial analysis, GIS processing services find the geo-features to be controlled. The control command and new state are assigned to the related geo-feature. The state is expressed by the characteristics.
- The control command and new state of the geo-feature are mapped to the corresponding controlling services.
- These controlling services adjust the state of the corresponding geo-entity.

From the information flow and the classification of the IoT service, the information flow in GIS-oriented IoT service application can be classified. The paper [19] described three types of information flow including sensing flow, controlling flow, business flow. We refer this classification, but we have different interpretations. GIS-oriented IoT service application consists of the sensing flow which is from geo-entity to geo-feature, the controlling flow which is from geo-feature to geo-entity and business flow which can process the geo-features. It is mainly the geo-feature which flows in the GIS-oriented IoT service application.

- **Sensing Flow:** The sensor device senses the information of geo-entity. The sensing service shares this information as the characteristic of the geo-feature to GIS processing service for further business analysis.
- **Business Flow:** GIS processing services receive the input information, use all kinds of spatial analysis methods to realize the business logic. A result is obtained after these processing. The result may contain a geo-feature with control commands.
- **Control Flow:** According to the control commands of the output geo-feature, the related controlling services adjust the states of the corresponding geo-entity.

These three kinds of information flows are closely related to each other. They constitute the information flow loop between the GIS-oriented IoT service application and the geographical environment. These flows complete the sense and control of geographic environment by the entire GIS-oriented IoT service application.

## 4. GIS-Oriented IoT Service Application Modeling

It can be seen from the information flow that GIS-oriented IoT service application uses the spatial analysis ability of GIS to process the information provided by the IoT service, and then output the result for business application. In the whole process, the sensing service and the GIS data service play the data provider role, and GIS processing service plays the role of spatial analysis and data processing. After data processing, the controlling service plays the role of adjusting the state of geo-entity. So the GIS-oriented IoT services application modeling is the process of composing all these services together to achieve the business goals.

We regard the whole business process as a sequence of service composition. When the user sends a request, the application system calls the first service; the first service calls the second service; the second service calls the third service, and so on. The output of the former service can provide the input for the latter service, or the former service is the precondition of the latter service. The sensing service and the GIS data service only provide data for the business process. There cannot be any other services before these two kinds of services, that is, these two kinds of services cannot call other services and they can only be called. Similarly, controlling services can only be called and cannot call other services. So in the following discussion, we directly regard the sensing service and GIS data service as the input of other processing service, and the controlling IoT service as the output of other service.

**Figure 5:**
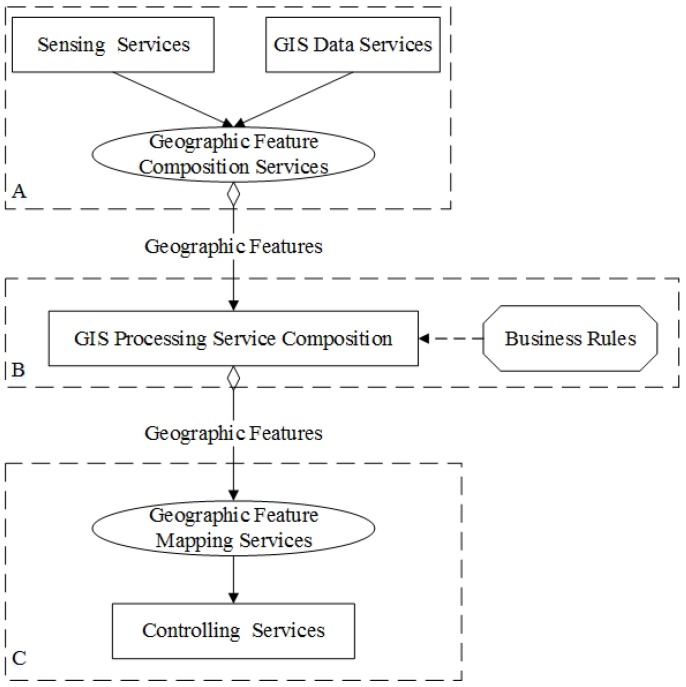
Tasks of GIS-oriented IoT service application modeling.

The service composition of GIS-oriented IoT service application can be divided into three tasks (Figure 5):

- **Sensing Service Composition.** The sensing service and the related GIS data service provide the input for Geo-feature composition service (GFCS). GFCS is an assisted service which can integrate the information from the sensing service and the GIS data service to a geo-feature. Here the sensing service and the GIS data service must be these services which describe the same geo-entity. After the data operation GFCS outputs the corresponding geo-feature.
- **GIS Processing Service Composition.** According to the business rule, the related GIS processing services are composed to process the input geo-feature, and then output business results including geo-feature with control commands.
- **Controlling Service Mapping.** The geo-feature mapping service (GFMS) receives the input geo-feature from the GIS processing service and maps the control commands to the corresponding controlling services and triggers these services to control the corresponding geo-entity.

Here we introduce a kind of service algebra, and then use the service algebra and CPN to discuss these three tasks. Service algebra focuses on describing what services are involved and what kind of composition relations are among these services. CPN focuses on describing what services are involved and the their composition process. service algebra is more concise, but CPN can describe the dynamic process.

### 4.1. Service Algebra

GIS-oriented IoT service application consists of the sensing service, the GIS data service, the GIS processing service, the controlling service and two assisted services including GFCS, GFMS. The Sensing service composition involves the sensing service, the GIS data service and GFCS. The controlling service mapping involves the controlling services and GFMS. And the way of GIS processing service composition is decided by the business rule. According to GIS-oriented IoT services application and the paper [49], we defined the service algebra for service composition. Based on service algebra, we can discuss more easily what services are involved and what kind of composition relations are among these services.

**Definition** **7.** **Service Algebra.** Multi atomic services can be composed to a complex service, and the complex service can be composed to a more complex service. This service composition process is called service operation. Service algebra is the operation rule for service composition. Service algebra is defined by the symbol like BNF. Let $GS_{1}$ and $GS_{2}$ are two services to be composed, GS is the composed service. The service algebra operations are as follows:

*$GS::=GS_{1}⊙GS_{2}\cup GS_{1}⊕GS_{2}\cup GS_{1}∥GS_{2}\cup \mu GS_{1}\cup GS_{1}⊢GS_{2}\cup GS_{1}⊣GS_{2}$*

**Figure 6:**
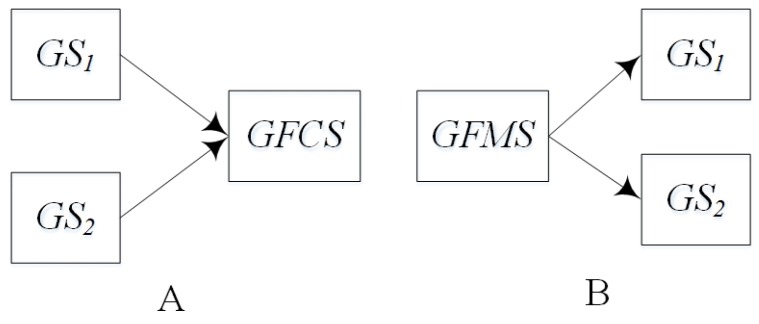
The composition and mapping of GIS-oriented IoT service application.

(1) *$GS_{1}⊙GS_{2}$ denotes that $GS_{1}$ and $GS_{2}$ are executed sequentially, i.e., first $GS_{1}$, then $GS_{2}$.* ⊙ *denotes the sequential operator.*
(2) *$GS_{1}⊕GS_{2}$ denotes that $GS_{1}$ and $GS_{2}$ are executed alternatively, that is, both $GS_{1}$ and $GS_{2}$ are not executed at the same time.* ⊕ *denotes the alternative operator.*
(3) $GS_{1}∥GS_{2}$ denotes that $GS_{1}$ and $GS_{2}$ are executed parallel, ∥ denotes the parallel operator.
(4) $\mu GS_{1}$ denotes the iterative operation, that is, $GS_{1}$ is executed repeatedly. μ denotes the iterative operator.
(5) *$GS_{1}⊢GS_{2}$ and $GS_{1}⊣GS_{2}$ are specially designed for the GIS-oriented IoT service. IoT services consist of the sensing service and the controlling service. The sensing service can be regard as a kind of data service. When multi data services provide input for another service, operator* ⊢ *can be used. For example, in Figure 6A denotes that $GS_{1}$ and $GS_{2}$ provide input for geo-feature composition service GFCS, i.e., $GS_{1}⊢GS_{2}$. Figure 6B denotes that through geo-feature mapping service GFMS, the control commands are mapped to the corresponding controlling services $GS_{1}$ and $GS_{2}$, i.e., $GS_{1}⊣GS_{2}$.*

The above mentioned service composition algebra operation are all closed operations [48] which can ensure that the result after service composition is still a service. The composed services can still participate in the services composition through service algebra in order to build services with more functionality and more complex structure. The properties of service algebra are summarized below.

(1) Services Commutative Law
  - $GS_{1}⊕GS_{2}=GS_{2}⊕GS_{1}$
  - $GS_{1}∥GS_{2}=GS_{2}∥GS_{1}$
  - $GS_{1}⊢GS_{2}=GS_{2}⊢GS_{1}$
  - $GS_{1}⊣GS_{2}=GS_{2}⊣GS_{1}$
(2) Services Associative Law
  - $\left(GS_{1}⊙GS_{2}\right)⊙GS_{3}=GS_{1}⊙\left(GS_{2}⊙GS_{3}\right)$
  - $\left(GS_{1}⊕GS_{2}\right)⊕GS_{3}=GS_{1}⊕\left(GS_{2}⊕GS_{3}\right)$
  - $\left(GS_{1}∥GS_{2}\right)∥GS_{3}=GS_{2}∥\left(GS_{2}∥GS_{3}\right)$
  - $\left(GS_{1}⊢GS_{2}\right)⊢GS_{3}=GS_{1}⊢\left(GS_{2}⊢GS_{3}\right)$
  - $\left(GS_{1}⊣GS_{2}\right)⊣GS_{3}=GS_{1}⊣\left(GS_{2}⊣GS_{3}\right)$
(3) Services Distributive Law
  - $GS_{1}⊙\left(GS_{2}⊕GS_{3}\right)=\left(GS_{1}⊙GS_{2}\right)⊕\left(GS_{1}⊙GS_{3}\right)$
  - $\left(GS_{1}⊕GS_{2}\right)⊙GS_{3}=\left(GS_{1}⊙GS_{3}\right)⊕\left(GS_{2}⊙GS_{3}\right)$
  - $GS_{1}∥\left(GS_{2}⊕GS_{3}\right)=\left(GS_{1}∥GS_{2}\right)⊕\left(GS_{1}∥GS_{3}\right)$
  - $\left(GS_{1}⊕GS_{2}\right)∥GS_{3}=\left(GS_{1}∥GS_{3}\right)⊕\left(GS_{2}∥GS_{3}\right)$
  - $GS_{1}⊢\left(GS_{2}⊕GS_{3}\right)=\left(GS_{1}⊢GS_{2}\right)⊕\left(GS_{1}⊢GS_{3}\right)$
  - $\left(GS_{1}⊕GS_{2}\right)⊢GS_{3}=\left(GS_{1}⊢GS_{3}\right)⊕\left(GS_{2}⊢GS_{3}\right)$
  - $GS_{1}⊙\left(GS_{2}∥GS_{3}\right)=\left(GS_{1}⊙GS_{2}\right)∥\left(GS_{1}⊙GS_{3}\right)$
  - $\left(GS_{1}∥GS_{2}\right)⊙GS_{3}=\left(GS_{1}⊙GS_{3}\right)∥\left(GS_{2}⊙GS_{3}\right)$
  - $GS_{1}⊢\left(GS_{2}∥GS_{3}\right)=\left(GS_{1}⊢GS_{2}\right)∥\left(GS_{1}⊢GS_{3}\right)$
  - $\left(GS_{1}∥GS_{2}\right)⊢GS_{3}=\left(GS_{1}⊢GS_{3}\right)∥\left(GS_{2}⊢GS_{3}\right)$

### 4.2. Sensing Service Composition

**Figure 7:**
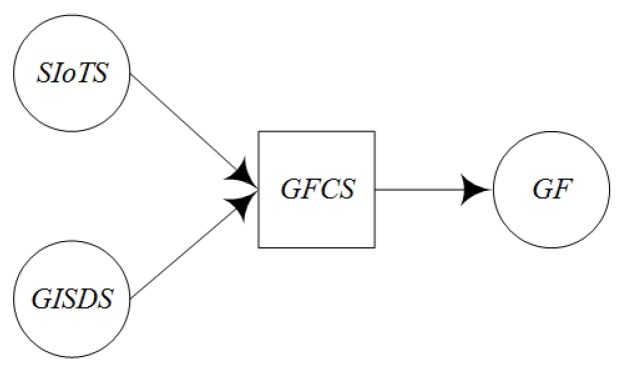
Sensing IoT service composition.

The geo-entity in the real world can be mapped to the geo-feature in the information world, and the characteristics of the geo-entity are mapped to the relevant characteristics of the corresponding geo-feature. The characteristics of geo-entity in the real world are described by the sensed information provided by the sensing service. We can integrate the sensed information of a geo-entity to a geo-feature which can be used for GIS processing service. Sometimes an existing GIS data service can also be involved in this integration. The process of geo-feature combination is called sensing service composition. This process is done by the assisted Geo-feature Composition Service (GFCS) (Figure 7).

**Definition** **8.** ***Sensing Service Composition:** Sensing service composition can be expressed as the following:*

(1) $$\begin{array}{cc} SIoTSC::= & SIoTS_{1}⊢SIoTS_{2}⊢...⊢SIoTS_{n}⊢GISDS⊙GFCS\\ = & \left(SSID,URL,SCS,QoS,SD,SSCPN\right) \end{array}$$

*where:*

(1) $SIoTS_{n}$ is the sensing service, GISDS is the GIS data service which is involved in the composition of geo-feature. GISDS is optional. GFCS is the service which compose $SIoTS_{n}$ and GISDS to geo-feature.
(2) SSID is the unique identifier of this composed service.
(3) URL is the invocation of the composed service.
(4) SCS is a set of its component services including $SIoTS_{n}$, GFCS, GISDS.
(5) QoS is the description of the quality of service.
(6) SD is the semantic function description.
(7) *SSCPN=(Σ,P,T,F,C,G,E,I) where:*
  - Σ *is a color set. It denotes the geo-feature type, data service type and other information type. The definition of colors includes:*
    Color SIoTS: the input sensing service,
    Color GISDS: the input GIS data service,
    Color GFT: the output geo-feature.
  - P is a finite set of places. P={i,o}, i is the input place set, o is the output place set. Type(i)∈{SIoTS,GISDS},Type(o)=GFT. Because there is no service calling the data service so ${}^{•}i=\emptyset$. Because the goal of the sensing service composition is to provide the input for the next process, so $o^{•}\neq \emptyset .$
  - T specially denotes the GFCS (geo-feature composition service) which composes multi sensing services to geo-feature. T consist of two actions including: (1) the composition action, which composes the information from the sensing service and the GIS data service to the geo-feature; (2) the action of completing data. The device service has instability. Some services may be suddenly failed and could not provide services. Then it is needed to complete the lost data. There are two ways to complete the lost data. One is to interpolate the information of the adjacent sensors in geographical space through spatial analysis. The other is to use the historical data of the service and the context.
  - F is arc set, F={(i,T),(T,o)}.
  - E is arc function set, E:F→E(f),E={E(i,T)=i,E(T,o)=o}.
  - C is color function, C={C(i)∈SIoTS∪GISDS,C(o)=GFT}.
  - G is the guard function which is defined according to the specific application scenarios. G can determine whether the service could be executed.
  - I is the initialization function which can confirm the initial conditions, I:P→Σ is the initial mark $M_{S}$ produced by the color value of every place. $\forall p\in P:Type\left(I\left(p\right)\right)=C{\left(p\right)}_{M_{S}}\wedge p\notin i\to I\left(p\right)\;=\;\emptyset .$

### 4.3. GIS Processing Service Composition

In GIS-oriented IoT service application, it is mainly GIS processing service that analyzes and processes the information provided by sensing services and other related GIS data services. However, a single GIS processing service cannot meet the complex requirements of the business, so it is necessary to compose multiple GIS processing services together to achieve business goals.

GIS processing atomic services can be composed to a complex service, and complex services can be composed to a more complex service. Service composition can be described by service algebra. We will discuss four kinds of service algebra including the sequential operation, the alternative operation, the parallel operation and the iteration operation.

Let $PS_{1},PS_{2}$ are two GIS processing services to be composed.

$PS_{1}=\left(PSID_{1},URL_{1},QoS_{1},SD_{1},PSCPN_{1}\right),$

$PS_{2}=\left(PSID_{2},URL_{2},QoS_{2},SD_{2},PSCPN_{2}\right).$

$i_{1},o_{1},i_{2},o_{2}$ is the input places and output places of $PS_{1}$ and $PS_{2}$.

**Figure 8:**
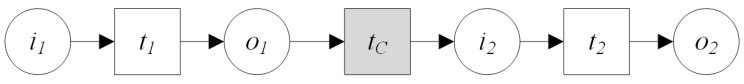
Sequential operation.

**Definition** **9.** ***Sequential Operation:** The sequential operation is that two services are executed in a predetermined order, and the input of the latter can be obtained from the output of the former service. The sequential operation (Figure 8) of two GIS processing services $PS_{1},PS_{2}$ can be described as:*

(2) $$\begin{array}{cc} GPSC::= & PS_{1}⊙PS_{2}\\ = & \left(PSID,URL,PCS,QoS,SD,PSCPN\right) \end{array}$$

*where:*

(1) PSID is the unique identifier of this new composed processing service.
(2) URL is the invocation of the composed service.
(3) PCS is a set of its component services including $PS_{1}$, $PS_{2}$.
(4) QoS is the description of the quality of service.
(5) SD is the semantic function description.
(6) *PSCPN=(Σ,P,T,F,C,G,E,I) where:*
  - $\Sigma =\Sigma_{1}\cup \Sigma_{2}$
  - $P=P_{1}\cup P_{2}\cup \left\{i_{1},i_{2},o_{1},o_{2}\right\}$. Let i is the input set of the new service, o is the output set of the new service, then $\left\{i,o\right\}\subseteq P,i=i_{1},o=o_{2}$. The relations of $o_{1}$ and $i_{2}$ consist of four kinds including exact,plugin,subsume,overlap. exact denotes $o_{1}=i_{2},$ that is, the output of $PS_{1}$ is the input of $PS_{2}$ and $PS_{1}$ can meet the requirements of $PS_{2}$. plugin denotes $o_{1}⊇i_{2}$, that is, the output of PS1 is more than the input of $PS_{2}$ and $PS_{1}$ can meet the requirements of $PS_{2}$. subsume denotes $o_{1}\subseteq i_{2}$, that is, the output of $PS_{1}$ is less than the input of $PS_{2}$ and $PS_{1}$ cannot meet the requirements of $PS_{2}$; $PS_{2}$ need the input from other services. overlap denotes $\left(o_{1}\cup i_{2}\right)\setminus \left(o_{1}\cap i_{2}\right)\neq \emptyset$, that is, the output of $PS_{1}$ is overlap with the input of $PS_{2}$, and $PS_{1}$ cannot meet the requirements of $PS_{2}$; $PS_{2}$ need the input from other services.
  - $T=T_{1}\cup T_{2}\cup \left\{t_{1},t_{2},t_{C}\right\},t_{C}$ is the added control transition which is used to compose $PS_{1}$ and $PS_{2}$ to a new service.
  - $F=F_{1}\cup F_{2}\cup \left\{\left(i_{1},t_{1}\right),\left(t_{1},o_{1}\right),\left(o_{1},t_{C}\right),\left(t_{C},i_{2}\right)\left(i_{2},t_{2}\right),\left(t_{2},o_{2}\right)\right\}.$
  - $C=C_{1}\cup C_{2}\cup \left\{C\left(i_{1}\right),C\left(o_{1}\right),C\left(i_{2}\right),C\left(o_{2}\right)\right\}.$
  - $G=G_{1}\cup G_{2}\cup G\left(t_{C}\right).$
  - $E=E_{1}\cup E_{2}\cup \left\{E\left(i_{1},t_{1}\right),E\left(t_{1},o_{1}\right),E\left(o_{1},t_{C}\right),E\left(t_{C},i_{2}\right),E\left(i_{2},t_{2}\right),E\left(t_{2},o_{2}\right)\right\}$
  - I is initialization function, and has the same definition with the I in Definition 6.

**Figure 9:**
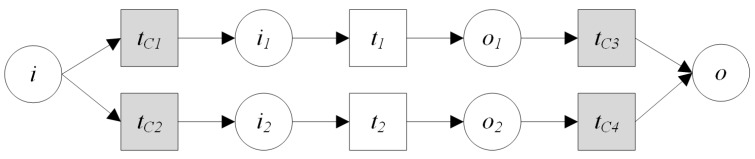
Alternative operation.

**Definition** **10.** ***Alternative Operation:** Both services $PS_{1}$ and $PS_{2}$ can meet the requirements of user, but only one of them can be executed. This kind of composition operation is called alternative operation. The alternative operation (Figure 9) of GIS processing services $PS_{1}$, $PS_{2}$ can be described as:*

(3) $$\begin{array}{cc} GPSC::= & PS_{1}⊕PS_{2}\\ = & \left(PSID,URL,PCS,QoS,SD,PSCPN\right) \end{array}$$

*where:*

(1) PSID is the unique identifier of this new composed processing service.
(2) URL is the invocation of the composed service.
(3) PCS is a set of its component services including $PS_{1}$, $PS_{2}$.
(4) QoS is the description of the quality of service.
(5) SD is the semantic function description.
(6) *PSCPN=(Σ,P,T,F,C,G,E,I) where:*
  - $\Sigma =\Sigma_{1}\cup \Sigma_{2}$
  - $P=P_{1}\cup P_{2}\cup \left\{i,o,i_{1},i_{2},o_{1},o_{2}\right\}.$ Let i is the input set of the new service, o is the output set of the new service, then $i_{1}\subseteq i$ or $i_{2}\subseteq i,o=o_{1}\cap o_{2}.$
  - $T=T_{1}\cup T_{2}\cup \left\{t_{1},t_{2},t_{C1},t_{C2},t_{C3},t_{C4}\right\}.t_{C1},t_{C2},t_{C3},t_{C4}$ are the added control transitions which are used to compose $PS_{1}andPS_{2}$ to a new service.
  - $F=F_{1}\cup F_{2}\cup \left\{\left(i,t_{C1}\right),\left(i,t_{C2}\right),\left(t_{C1},i_{1}\right),\left(t_{C2},i_{2}\right),\left(i_{1},t_{1}\right),\left(i_{2},t_{2}\right),\left(t_{1},o_{1}\right),\left(t_{2},o_{2}\right),\left(o_{1},t_{C3}\right),\left(o_{2},t_{C4}\right),\left(t_{C3},o\right),\left(t_{C4},o\right)\right\}$.
  - $C=C_{1}\cup C_{2}\cup \left\{C\left(i\right),C\left(o\right),C\left(i_{1}\right),C\left(o_{1}\right),C\left(i_{2}\right),C\left(o_{2}\right)\right\}.$
  - $G=G_{1}\cup G_{2}\cup \left\{G\left(t_{1}\right),G\left(t_{2}\right),G\left(t_{C1}\right),G\left(t_{C2}\right),G\left(t_{C3}\right),G\left(t_{C4}\right)\right\}.$
  - $E=E_{1}\cup E_{2}\cup \left\{E\left(i,t_{C1}\right),E\left(i_{1},t_{C2}\right),E\left(t_{C1},i_{1}\right),E\left(t_{C2},i_{2}\right),E\left(i_{1},t_{1}\right),E\left(i_{2},t_{2}\right),E\left(t_{1},o_{1}\right),E\left(t_{2},o_{2}\right),E\left(o_{1},t_{C3}\right),E\left(o_{2},t_{C4}\right),E\left(t_{C3},o\right),E\left(t_{C4},o\right)\right\}.$
  - I is initialization function, and has the same definition with the I in Definition 6.

**Figure 10:**
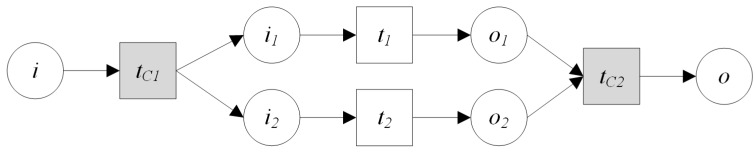
Parallel operation.

**Definition** **11.** ***Parallel Operation:** If two services are all needed to meet the requirements of user and these two services are independent with each other. Service composition would execute them concurrently. This composition operation is called parallel operation. The parallel operation (Figure 10) of GIS processing services $PS_{1}$, $PS_{2}$ can be described as:*

(4) $$\begin{array}{cc} GPSC::= & PS_{1}∥PS_{2}\\ = & \left(PSID,URL,PCS,QoS,SD,PSCPN\right) \end{array}$$

*where:*

(1) PSID is the unique identifier of this new composed processing service.
(2) URL is the invocation of the composed service.
(3) PCS is a set of its component services including $PS_{1}$, $PS_{2}$.
(4) QoS is the description of the quality of service.
(5) SD is the semantic function description.
(6) *PSCPN=(Σ,P,T,F,C,G,E,I) where:*
  - $\Sigma =\Sigma_{1}\cup \Sigma_{2}$.
  - $P=P_{1}\cup P_{2}\cup \left\{i,o,i_{1},i_{2},o_{1},o_{2}\right\},$ Let i is the input set of the new service, o is the output set of the new service, then $i=i_{1}\cup i_{2},o=o_{1}\cup o_{2}.$
  - $T=T_{1}\cup T_{2}\cup \left\{t_{1},t_{2},t_{C1},t_{C2}\right\}.t_{C1},t_{C2}$ are the added control transitions which are used to compose $PS_{1}andPS_{2}$ to a new service.
  - $F=F_{1}\cup F_{2}\cup \left\{\left(i,t_{C1}\right),\left(t_{C1},i_{1}\right),\left(t_{C1},i_{2}\right),\left(i_{1},t_{1}\right),\left(i_{2},t_{2}\right),\left(t_{1},o_{1}\right),\left(t_{2},o_{2}\right),\left(o_{1},t_{C2}\right),\left(o_{2},t_{C2}\right),\left(t_{C2},o\right)\right\}$.
  - $C=C_{1}\cup C_{2}\cup \left\{C\left(i\right),C\left(o\right),C\left(i_{1}\right),C\left(o_{1}\right),C\left(i_{2}\right),C\left(o_{2}\right)\right\}.$
  - $G=G_{1}\cup G_{2}\cup \left\{G\left(t_{1}\right),G\left(t_{2}\right),G\left(t_{C1}\right),G\left(t_{C2}\right)\right\}.$
  - $E=E_{1}\cup E_{2}\cup \left\{E\left(i,t_{C1}\right),E\left(t_{C1},i_{1}\right),E\left(t_{C1},i_{2}\right),E\left(i_{1},t_{1}\right),E\left(i_{2},t_{2}\right),E\left(t_{1},o_{1}\right),E\left(t_{2},o_{2}\right),E\left(o_{1},t_{C2}\right),E\left(o_{2},t_{C2}\right),E\left(t_{C2},o\right)\right\}.$
  - I is initialization function, and has the same definition with the I in Definition 6.

**Figure 11:**
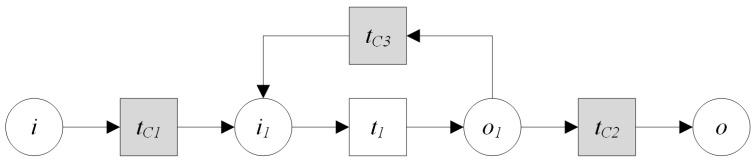
Iteration operation.

**Definition** **12.** ***Iteration Operation:** If a service is called repeatedly in a process of service composition, this kind of composition operation is called iteration operation. The iteration operation (Figure 11) of GIS processing service $PS_{1}$ can be described as:*

(5) $$\begin{array}{cc} GPSC::= & \mu PS_{1}\\ = & \left(PSID,URL,PCS,QoS,SD,PSCPN\right) \end{array}$$

*where:*

(1) PSID is the unique identifier of this new composed processing service.
(2) URL is the invocation of the composed service.
(3) PCS is a set of its component services including several $PS_{1}$.
(4) QoS is the description of the quality of service.
(5) SD is the semantic function description.
(6) *PSCPN=(Σ,P,T,F,C,G,E,I) where:*
  - $\Sigma =\Sigma_{1}$.
  - $P=P_{1}\cup \left\{i,o,i_{1},o_{1}\right\},$ Let i is the input set of the new service, o is the output set of the new service, then $i_{1}=i\cup o_{1},o\subseteq o_{1}.$
  - $T=T_{1}\cup \left\{t_{1},t_{C1},t_{C2},t_{C3}\right\}.t_{C1},t_{C2},t_{C3}$ are the added control transitions which are used to execute $PS_{1}$ repeatedly.
  - $F=F_{1}\cup \left\{\left(i,t_{C1}\right),\left(t_{C1},i_{1}\right),\left(i_{1},t_{1}\right),\left(t_{1},o_{1}\right)\left(o_{1},t_{C3}\right),\left(t_{C3},i_{1}\right),\left(o_{1},t_{C2}\right),\left(t_{C2},o\right)\right\}$.
  - $C=C_{1}\cup \left\{C\left(i\right),C\left(o\right),C\left(i_{1}\right),C\left(o_{1}\right)\right\}.$
  - $G=G_{1}\cup \left\{G\left(t_{1}\right),G\left(t_{C1}\right),G\left(t_{C2}\right),G\left(t_{C3}\right)\right\}.$
  - $E=E_{1}\cup \left\{E\left(i,t_{C1}\right),E\left(t_{C1},i_{1}\right),E\left(i_{1},t_{1}\right),E\left(t_{1},o_{1}\right),E\left(o_{1},t_{C3}\right),E\left(t_{C3},i_{1}\right),E\left(o_{1},t_{C2}\right),,E\left(t_{C2},o\right)\right\}.$
  - I is initialization function, and has the same definition with the I in Definition 6.

### 4.4. Controlling Service Mapping

After the business analysis performed by GIS processing services, if the business goals need to change the states of some geo-entities, then GIS processing services would output geo-features with control commands. In Definition 2, we defined a formal description of the geo-feature, each of which has a set of permitted operations. These operations are corresponding to the controlling services in GIS-oriented IoT service application. Each control command can be executed by a controlling service to change the state of the corresponding geo-entity. We need to map the control command to the corresponding controlling service and trigger this service. There is a spatial service which is called GFMS (Geo-feature mapping service) to execute this functionality. The process of mapping and triggering is called controlling service mapping.

For example, a GIS processing service outputs a geo-feature GF with a control command set OP. $op_{1},op_{2}\in OP.$ $op_{1},op_{2}$ are corresponding to the controlling services $s_{1},s_{2}.$ $s_{1},s_{2}$ are triggered to control the state of geo-entity.

The controlling service mapping consists of the geo-feature mapping service, the controlling service. It receives the geo-feature with control commands from the former GIS processing service.

**Figure 12:**
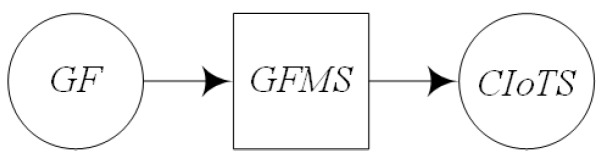
Controlling IoT services mapping.

**Definition** **13.** ***Controlling Service Mapping:** A controlling service mapping (Figure 12) can be expressed as:*

(6) $$\begin{array}{cc} CIoTSM::= & GFMS⊙CIoTS_{1}⊣CIoTS_{2}⊣...⊣CIoTS_{n}\\ = & \left(CSID,URL,CCS,QoS,SD,CSCPN\right) \end{array}$$

*where:*

(1) GFMS is the service which maps the control commands from geo-feature to the corresponding controlling IoT services. $CIoTS_{n}$ is controlling service which can change the state of geo-entity.
(2) CSID is the unique identifier of this new composed mapping service.
(3) URL is the invocation of the composed service.
(4) CCS is a set of its component services including $GFMS,CIoTS_{n}$.
(5) QoS is the description of the quality of service.
(6) SD is the semantic function description.
(7) *PSCPN=(Σ,P,T,F,C,G,E,I) where:*
  - Σ *is a color set, It denotes the input geo-feature and the output controlling service. The definition of colors includes:*
    Color CIoTS: the output controlling service,
    Color GFT: the input geo-feature.
  - P is a finite set of places. $P=\left\{i,o\right\}{,}^{•}i=\emptyset ,o^{•}\neq \emptyset .$ i is the input place set, o is the output place set. Type(i)=GFT,Type(o)=CIoTS.
  - T specially denotes the GFMS (geo-feature mapping service) which maps the control commands of the input geo-feature to the controlling services and trigger them. T consists of three actions including: (1) the action which maps the control commands to the controlling services; (2) the action which triggers these controlling services; (3) the action which solves the problem of failed service call caused by the instability of IoT services.
  - F is arc set, F={(i,T),(T,o)}.
  - C is color function, C={C(i)=GFT,C(o)=CIoTS}.
  - G is the guard function which is defined according to the specific application scenarios. G can determine whether the service could be executed.
  - E is arc function set, E:F→E(f).
  - I is initialization function, and has the same definition with the I in Definition 6.

### 4.5. GIS-Oriented IoT Service Application Model

As is shown in Figure 5, GIS-oriented IoT service application modeling task includes three parts. The first part is sensing service composition which provide geo-features for the next step of GIS processing services. The second part is GIS processing service composition. GIS processing services are composed to process the input geo-features according to the business requirements. The third part is controlling services mapping which maps the control commands of geo-features to the corresponding controlling services and triggers them. Services of GIS-oriented IoT consist of the sensing service, the GIS data service, the GIS processing service, the controlling service and two assisted service including GFCS, GFMS. Therefore, the process of GIS-oriented IoT service application modeling is to composed these three tasks, and composed all these services to meet the requirements of user.

Let SIoTSC is sensing service composition, CIoTSC is controlling services mapping, and GPSC is GIS processing service composition. Then according to the above discuss, the following discuss the definition of GIS-oriented IoT service application.

**Definition** **14.** ***GIS-Oriented IoT Service Application Model:** GIS-oriented IoT service application can be described as:*

(7) $$\begin{array}{cc} GIoTSAM::= & SIoTSC⊙GPSC⊙CIoTSC\\ = & \left(GMID,URL,GCS,QoS,SD,GMCPN\right) \end{array}$$

*where: GMID is the unique identifier of this new composed service. URL is the invocation of the composed service. GCS is a set of its component services. QoS is the quality of the new service. SD is the semantic description of the new service.*

**Figure 13:**
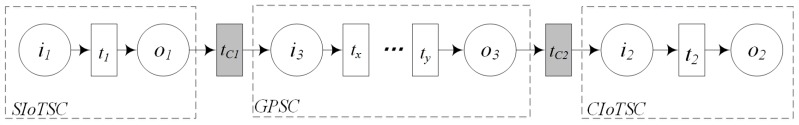
CPN model of GIS-oriented IoT service application.

GMCPN=(Σ,P,T,F,C,G,E,I) where:

(1) Σ *is the color set in GIS-oriented IoT service application. The definition of colors includes:*
  Color SIoTS: the input sensing IoT service,
  Color CIoTS: the output controlling IoT service,
  Color GISDS: the input GIS data services,
  Color GFT: the output geo-feature,
  Color STR: the input and output other information types, such as number, string.
(2) P is a finite set of places. Let i is the input places, o is the output places. $P=\left\{i,o\right\}\cup P_{SIoTSC}\cup P_{GPSC}\cup P_{CIoTSC}.Type\left(i\right)\in \left\{SIoTS,GFT,GISDS,STR\right\}.Type\left(o\right)\in \left\{CIoTS,GFT,STR\right\}{,}^{•}i=\emptyset ,o^{•}=\emptyset .$
(3) T is a finite set of transitions. There are two kinds of transitions in GIS-oriented IoT service application. One kind is the transition inside a service, which is called service transition $T_{S}$. It is the operation inside a service. The other kind is control transition $T_{C}$ which is the composition operation between services and used for the connections between services. $T=T_{S}\cup T_{C}$. In Figure 13, $T=T_{SIoTSC}\cup T_{GPSC}\cup T_{CIoTSC}\cup t_{C1}\cup t_{C2}.$
(4) F is a finite set of arc. F⊆P×T∪T×P. In Figure 13, $F=F_{SIoTSC}\cup F_{GPSC}\cup F_{CIoTSC}\cup \left\{\left(o_{1},t_{C1}\right),\left(t_{C1},i_{1}\right),\left(o_{3},t_{C2}\right),\left(t_{C3},i_{2}\right)\right\}.$
(5) C is a color function set, C:P→Σ. In Figure 13, $C=C_{SIoTSC}\cup C_{GPSC}\cup C_{CIoTSC}$.
(6) G is a guard function set, $G=G\left(T_{S}\right)\cup G\left(T_{C}\right).$
(7) E is an arc function set, In Figure 13, $E=E_{SIoTSC}\cup E_{GPSC}\cup E_{CIoTSC}\cup \left\{E\left(o_{1},t_{C1}\right),E\left(t_{C1},i_{1}\right),E\left(o_{3},t_{C2}\right),E\left(t_{C3},i_{2}\right)\right\}.$
(8) I is initialization function, and has the same definition with the I in Definition 6.

In GIS-oriented IoT service application, the geographical space and the information space are closely linked. Geo-entities and features become the important participants of business process. The staff can interact with these geo-entities, and can easily query and change their state or send control instructions.

## 5. Modeling Example

### 5.1. Description of Forest Fire Simulation Scenario

The IoT plays an important role in forest fire monitoring and prevention. The monitoring sensor equipment and firefighting equipment in the forest have obvious spatial distribution characteristics. So it is suitable for the model proposed in this paper.

**Figure 14:**
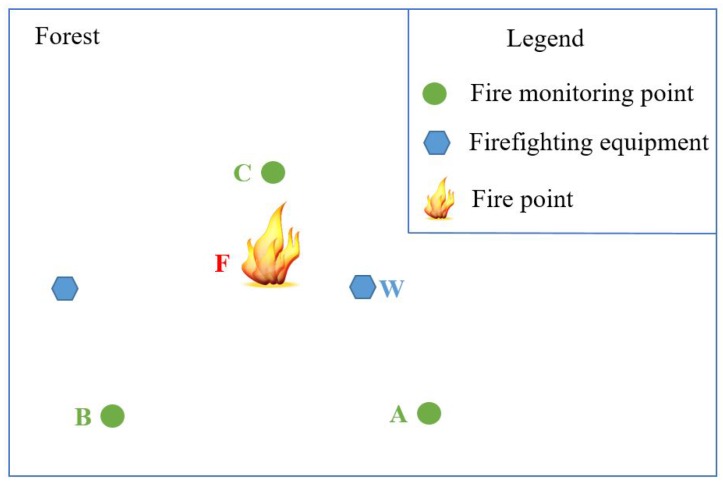
Sketch map of forest fire.

Figure 14 is the Sketch Map of Forest Fire. All the information in the map is assumed, such as map scale, the network of equipment layout and so on. All the assumed information is only for modeling schematic. The green points are fire monitoring points, the blue point are fire extinguishing equipments. we assume the extinguishing equipment is fixed water cannon. After triggering the automatic fire extinguishing process, the whole business process is as follows:

(1) According to the location of three sensing sensor A,B,C and the detected information of temperature, humidity, infrared, ultraviolet and smoke from A,B and *C*, the fire point location *F* is found by spatial analysis.
(2) According to the location of the fire point *F*, the nearest firefighting water cannon *W* is found by the buffer analysis and the nearest distance analysis.
(3) By the spatial analysis of the temperature, humidity and other information of three monitoring points A,B,C and the distance between the fire point *F* and the firefighting water cannon *W*, the adjustment information of water quantity, direction and angle of the firefighting water cannon is caculated.
(4) The adjustment information is used to adjust the firefighting water cannon *W*. The firefighting water cannon *W* is instructed to adjust the water quantity, direction and angle to put out fire.

### 5.2. Modeling

#### 5.2.1. Assumptions

**Figure 15:**
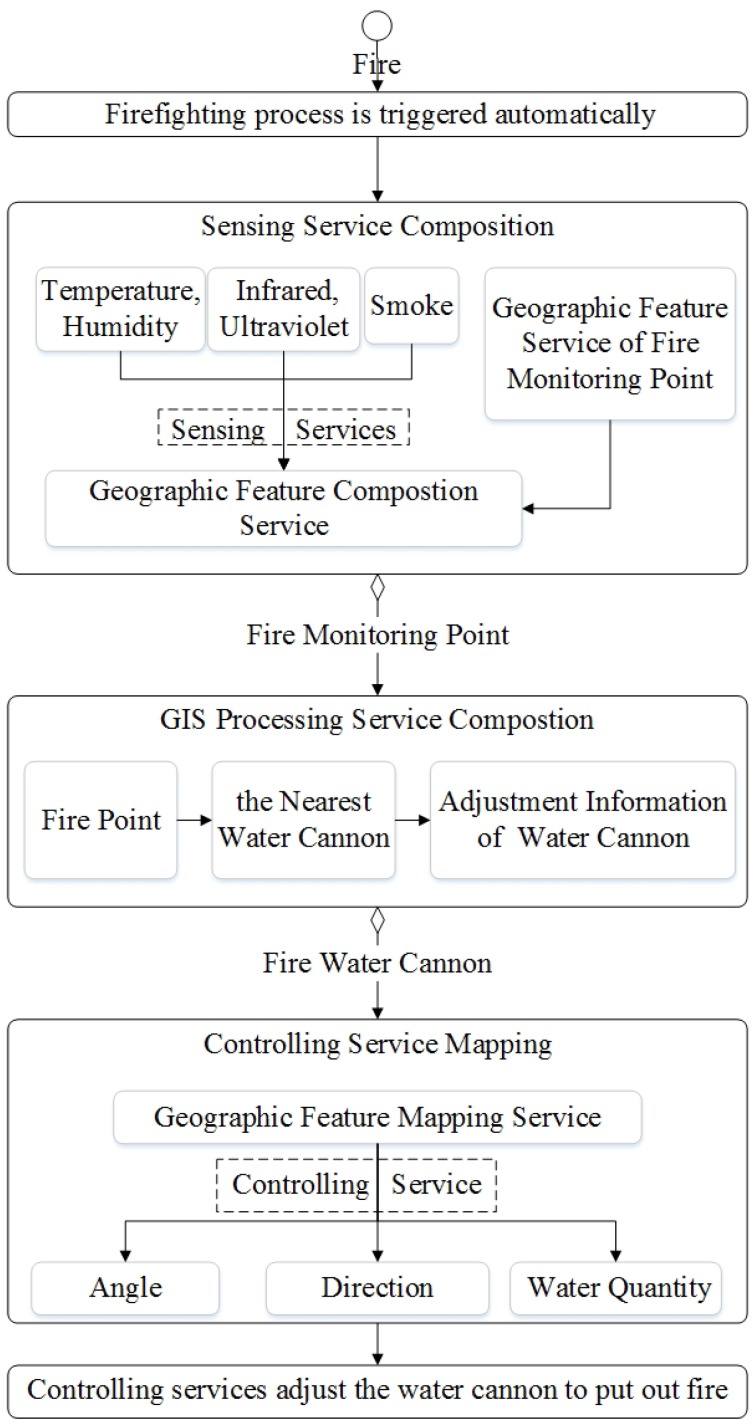
Automatic firefighting process.

According to the above automatic firefighting process, we analyze the simulation scenario and draw a firefighting flowchart (Figure 15). For this simulation scenario, we make the following assumptions for the convenience of modeling and describing the whole process:

(1) Each of the fire monitoring point and the firefighting water cannon is regarded as a geo-entity. There are two GIS data services for these two kinds of equipment. However, the geo-feature set in the GIS data service of fire monitoring point only has some basic attributes such as location coordinate, ownership, installation date and so on. The attributes of temperature and humidity and other real-time information must be obtained from the related device service. The related GIS data services consist of the fire monitoring point service $DS_{fm}$ and the firefighting water cannon service $DS_{fd}$.
(2) Each fire monitoring point is equipped with temperature and humidity sensor, infrared and ultraviolet sensor, smoke sensor. These devices can provide temperature and humidity service ($S_{th}$), infrared and ultraviolet service ($S_{iu})$, smoke service ($S_{sm}$). Then in the figure, the services from fire monitoring point A,B,C include $A\_S_{th}$, $A\_S_{iu}$, $A\_S_{sm}$, $B\_S_{th}$, $B\_S_{iu}$, $B\_S_{sm}$, $C\_S_{th}$, $C\_S_{iu}$, $C\_S_{sm}.$
(3) There are two assisted services: geo-feature composition service GFCS and geo-feature mapping service GFMS.
(4) The firefighting water cannon can provide controlling services, that is, the controlling services can be called to control the state of this firefighting water cannon to put out fire automatically. The states including angle ($S_{a}$), direction ($S_{d}$) and water quality ($S_{w}$) of this firefighting water cannon *W* are controlled by three controlling device services $W\_S_{a},W\_S_{d},W\_S_{w}.$
(5) The related GIS processing services consist of fire point information analysis service $PS_{cf}$ which is used to find the fire point, buffer analysis and nearest distance analysis service $PS_{ba}$ which is used to find the nearest firefighting water cannon, the state analysis service of firefighting water cannon $PS_{fca}$, which is used to calculate the adjustment information of the firefighting water cannon.

#### 5.2.2. Service Algebra Modeling

Based on above assumption, we begin to model this example using the modeling method proposed by this paper.

(1) **Sensing service composition.** The sensing services ($A\_S_{th}$, $A\_S_{iu}$, $A\_S_{sm}$, $B\_S_{th}$, $B\_S_{iu}$, $B\_S_{sm}$, $C\_S_{th}$, $C\_S_{iu}$, $C\_S_{sm}$), the related GIS data service $\left(DS_{fm}\right)$ about the same geo-entities (A,B,C) and the geo-feature composition service (GFCS) are composed to output the geo-features of fire monitoring point. The expression of service algebra is:
  $S_{gfc}::=\left(A\_S_{th}⊢A\_S_{iu}⊢A\_S_{sm}⊢B\_S_{th}⊢B\_S_{iu}⊢B\_S_{sm}⊢C\_S_{th}⊢C\_S_{iu}⊢C\_S_{sm}\right)⊢DS_{fm}⊙GFCS$.
(2) **GIS processing service composition.** These three spatial analysis including fire point information analysis service $\left(PS_{cf}\right)$, buffer analysis service $\left(PS_{ba}\right)$ and the state analysis service of firefighting device $\left(PS_{fca}\right)$ are executed according to a certain order. The input of $PS_{ba}$ needs the output of $PS_{cf}$ and the GIS data service $GD\_S_{fd}$. The expression of service algebra is:
  $S_{gfsc}::=PS_{cf}⊢GD\_S_{fd}⊙PS_{ba}⊙PS_{fca}$.
(3) **Controlling service mapping.** The geo-feature mapping service (GFMS) maps the control commands (the adjustment information of angle, direction and water quality) in the geo-feature (firefighting water cannon *W*) to the related controlling services $\left(W\_S_{a},W\_S_{d},W\_S_{w}\right)$. The expression of service algebra is:
  $S_{gfd}::=GFMS⊙W\_S_{a}⊣W\_S_{d}⊣W\_S_{w}$.
(4) **GIS-oriented IoT service application model.** The above three parts are integrated to a GIS-oriented IoT service application model. The expression of service algebra is:
  $S::=S_{gfc}⊙S_{gfsc}⊙S_{gfd}::=\left(A\_S_{th}⊢A\_S_{iu}⊢A\_S_{sm}⊢B\_S_{th}⊢B\_S_{iu}⊢B\_S_{sm}⊢C\_S_{th}⊢C\_S_{iu}⊢C\_S_{sm}\right)⊢DS_{fm}⊙GFCS⊙PS_{cf}⊢GD\_S_{fd}⊙PS_{ba}⊙PS_{fca}⊙GFMS⊙W\_S_{a}⊣W\_S_{d}⊣W\_S_{w}$.

#### 5.2.3. CPN Modeling

**Figure 16:**
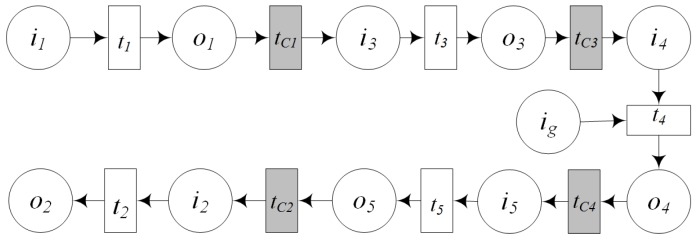
CPN model of the automatic firefighting process.

From the service algebra, we can know what services are involved and what kinds of relations between them, but we don’t know how they work together. In Figure 16, this firefighting example is modeled using CPN. From this model we can know how these services work together.

(1) **Sensing Service Composition.** $i_{1}$ is the input of GFCS, $o_{1}$ is the output of GFCS. $t_{1}$ is the internal operations of GFCS. $i_{1}=\{$$A\_S_{th}$, $A\_S_{iu}$, $A\_S_{sm}$, $B\_S_{th}$, $B\_S_{iu}$, $B\_S_{sm}$, $C\_S_{th}$, $C\_S_{iu}$, $C\_S_{sm}$}. $o_{1}$ is the composed geo-feature set including fire monitoring points A,B,C.
(2) **GIS Processing Service Composition.**
  **Finding the fire point.** $i_{3}$ is the input of $PS_{cf}$ and is the geo-feature set of fire monitoring points A,B,C; $o_{3}$ is the output of $PS_{cf}$ and is the geo-feature of fire point *F*; $t_{3}$ is the internal operations of $PS_{cf}$.
  **Finding the nearest firefighting water cannon.** $i_{4}$ is the input of $PS_{bf}$ and is the geo-feature of fire point *F*; $i_{g}$ is another input of $PS_{bf}$ and is GIS data service of firefighting water cannons; $o_{4}$ is the output of $PS_{bf}$ and is the geo-feature of the nearest firefighting water cannon *W* and fire point *F*; $t_{4}$ is the internal operations of $PS_{bf}$.
  **Calculating the adjustment information (control commands) of the water cannon.** $i_{5}$ is the input of $PS_{cfa}$, and is the geo-feature of fire point *F*; $o_{5}$ is the output of $PS_{cfa}$ and is the geo-feature of firefighting water cannon with control commands; $t_{5}$ is the internal operations of $PS_{cfa}$.
(3) **Controlling Service Mapping.** $i_{2}$ is the input of GFMS, and is geo-features with control commands; $o_{2}$ is the mapped controlling services. $t_{2}$ is the internal operation of GFMS which mapping the control commands to the corresponding controlling device services. $o_{2}=W\_S_{a},W\_S_{d},W\_S_{w}$.
(4) **Other Interpretation.** $t_{C1},t_{C2},t_{C3},t_{C4}$ are control transitions, which are used to compose the adjacent services, then the former service can provide the input for the latter service.

#### 5.2.4. Simulating by CPN Tools

CPN Tools is an effective tool for creating, editing, simulating, and analyzing Colored Petri nets.

When we are constructing a net using this tool, it features incremental syntax checking and code generation. CPN Tools can simulate untimed and timed nets fast and efficiently, generate and analyze full and partial state spaces.

According the above discuss, if the sensing services $(A\_S_{th}$, $A\_S_{iu}$, $A\_S_{sm}$, $B\_S_{th}$, $B\_S_{iu}$, $B\_S_{sm}$, $C\_S_{th}$, $C\_S_{iu}$, $C\_S_{sm})$ and corresponding GIS data service $\left(DS_{fm}\right)$ can provide the correct parameters, the tokens in $i_{1}$ are completed. Then under the initial mark, the transition GFCS is enabled by $M_{0}$, i.e., $\forall t\in T,M_{0}[t>$. After the transition GFCS is executed, the transition GFCS creates the fire point geo-features A,B,C which are the needed input conditions of the transition $PS_{cf}$. TC1 connects the input of $PS_{cf}$ and output of GFCS. Because the tokens in i3 are enough, so the transition $PS_{cf}$ is enabled at this time.

The transition $PS_{cf}$ consumes three tokens (the fire point geo-features A,B,C) from i3 and create one token which represents the fire point (FP). Then the place o3 contains one token which is the needed condition for the transition $PS_{b}a$. The transition tc3 is used to connect the service $PS_{cf}$ and $PS_{ba}$. So the input token and output token are the same.

The token which represents the fire point (FP) from transition $PS_{cf}$ and the token from ig are consumed by the transition $PS_{ba}$, and one token which represents the nearest firefighting water cannon is created. This token is needed for the transition $PS_{cfa}$. $PS_{cfa}$ needs one token to execute, and creates one token for the next transition. The consumed token represents the nearest firefighting water cannon, the created token represents the same water cannon with new controlling commands. The transition tc4 connects the service $PS_{cfa}$ and $PS_{ba}$.

The transition tc2 is used to connect the service $PS_{cfa}$ and GFMS. The transition GFMS consumes the token which represents the firefighting water cannon with new controlling command, and created three tokens which represent the controlling services.

Then, we can know that the model is valid and terminable. The CPN Tools can simulate the model and analyze its properties such as boundedness properties and liveness properties. The CPN model can express the composition logic of the composed services.

### 5.3. Performance of the Composition

The performance of Web service composition is very important for the real application. The performance of Web service composition is related to the method of combination and the performance of each composed service. The paper [63] proposed a service composition approach based on service clusters. This approach classified the composed services to service clusters for quick service composition. The paper [64] proposed a service composition method according to the quality of service (QoS) requirement for the service composition satisfying the Markov property.

**Figure 17:**
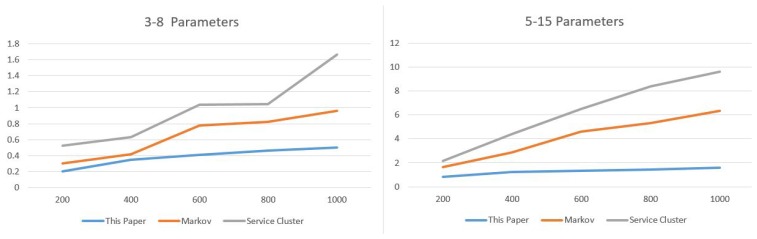
Comparison of execution time.

In this performance experiment, we designed 1000 GIS processing services and simulated 20 sensing services and 20 controlling services. Every service can composed to the other by some parameters. When there are 3–8 paramters, all these parameters can be composed to a geo-feature. When there are 5–15 parameters, these parameters can be composed to 2 geo-features. We used these three method for this experiment, and compared their execution time. The experiment result is shown in Figure 17. The horizontal coordinate represents the number of services. The vertical coordinate represents the execution time. The left part of the figure shows the results of 3–8 parameters, the right of the figure shows the result of 5–15 parameters. We can come to the conclusion that the method proposed by this paper has certain advantages.

**Figure 18:**
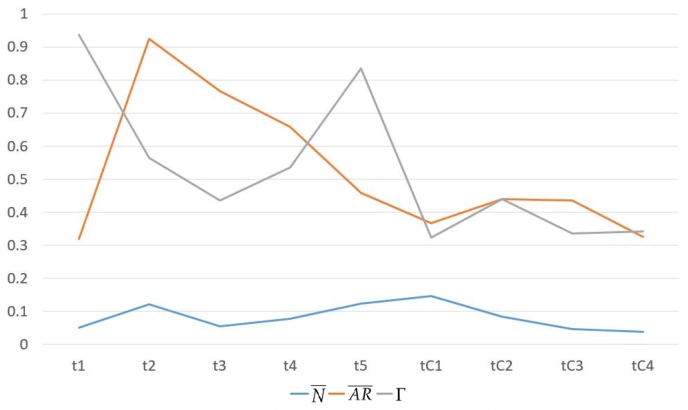
Performance parameter.

The performance of each composed service can influence the performance of the whole service composition. So, how to identify the performance of each service becomes very important. The paper [65] proposed a method BRF-WSCPAM to locate the performance bottleneck of the service composition and to evaluate the performance of each service. For more details about BRF-WSCPAM, please refer to the original document. Table 1 shows the configuration parameters. Table 2 shows the performance parameter calculated by BRF-WSCPAM. Γ denotes the bottleneck risk coefficient. The higher the coefficient, the higher the bottleneck risk. From Figure 18 and Table 2 we can see that the transition $t_{1}$ and $t_{5}$ cost more time than others. So the performance of the corresponding service is not good.

### 5.4. Compared with Activity Diagram Variants

In Section 2.2 we have introduced many service composition methods. The workflow-based composition method is one of the most important ways. Like Petri Nets, the UML activity diagrams [66] are graphical too. They use bubbles and arrows, are vendor-independent, so they can express most desirable routing constructs [67]. In this section, we will compare Petri Nets and Activity Diagram Variants in service composition modeling.

In the process of service composition, the key elements are the input and output data, the function of service and the connection of two services.

**Figure 19:**
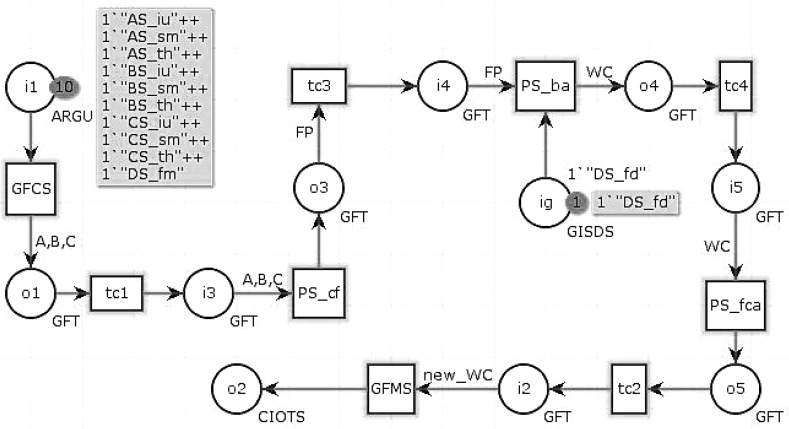
CPN model in CPN Tools.

**Figure 20:**
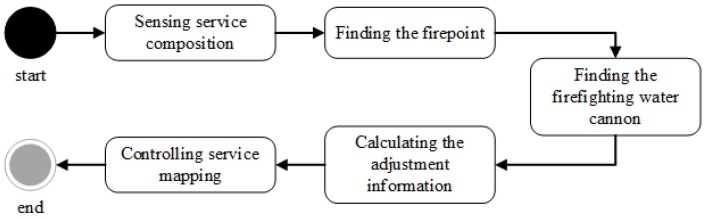
Activity diagram for service composition.

As is shown in Figure 19 and Figure 20, CPN model uses colored token to represent the input and output data of the composed services. The input and output relationship of two services is represented by consuming or creating of the token. The different data type is represented by the different color set. By the simulation of the model, we can see the dependency relation between the composed services. The activity diagram uses local variable to represent the input and output data of the composed services.

The activity diagram uses the activity to represent the function of the services and the connection of two services. CPN model uses transition to represent the function of the services and the connection of two services. The transition uses the the consuming or creating of the token to complete its internal function. The tokens given by the former transition determine whether the latter transition can be fired. However, from Figure 20 we do not see this relationship.

The activity diagram has full semantic expression ability, but it is more suitable for the workflow modeling, not for service composition. The Petri Nets also has stronger mathematical support, so it can model the service composition better.

### 5.5. Two Other Examples

#### 5.5.1. Example of Large Parking Lot

**Figure 21:**
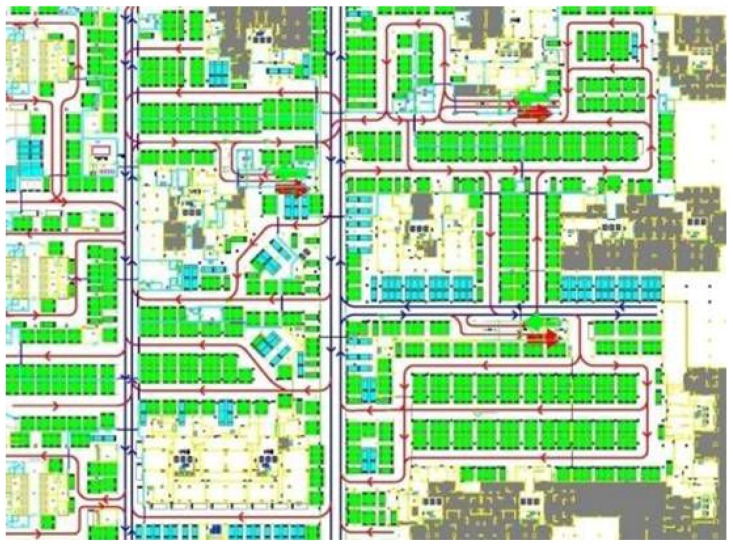
Parking lot of a large shopping mall. The green part is the parking space.

In some large parking lot, like the Figure 21, finding a suitable parking space is very difficult, especially when there are many cars. Then it is need the IoT and GIS technologies to solve this problem. Here we only consider the IoT service and GIS service, and simplify other aspects of the technical problems. The following is a brief description of the process of parking.

In the parking lot, we assume that there are three types of parking equipment including the license plate number identification equipment *A* at the entrance, the parking space navigating equipment *B* along the road in the parking lot and the equipment *C* which senses the parking state of every parking space. Each equipment has its own sensor. At the entrance of the parking lot, when there is a car coming into the parking lot, the equipment *A* identifies the license plate number of the car and sends the number to the GIS Service $PS_{ba}$ by the IoT sensing service $S_{ei}$. The service $PS_{ba}$ finds the nearest free parking space FP and sends FP to the service $PS_{sr}$. The service $PS_{sr}$ calculates the shortest route to FP and send the route to the service $PS_{ba}$ to find the equipments $B_{r}$ along this route. These equipments $B_{r}$ and the route are send to the service $PS_{nc}$ to calculate the navigating commands. Then the service $PS_{nc}$ sends these navigating commands and the license plate number of the car to the controlling service $CS_{ni}$ of every equipment $B_{r}$ along the shortest route. When the car entered the entrance of the parking lot, the nearest $B_{r}$ sensed its license plate number and shows the corresponding navigating command on the screen, for example, “The car of license plate number XXXX, please turn left”. After every navigating command of the equipment $B_{r}$, the car is parked in the parking space. The equipment *C* perceived the parked car, and sends the new state of this parking space to the GIS processing service $PS_{ps}$ to update parking space data. In the whole process, there are some other GIS data services like the road data $DS_{r}$, the equipment *B* data service $DS_{b}$. Table 3 shows the services in the parking lot and Table 4 shows the attachments of these services.

The following equation uses Service Algebra to model the parking process.

$$\begin{array}{c} S::=S_{ei}⊢DS_{a}⊙GFCS⊢DS_{p}⊙PS_{ba}⊙PS_{sr}⊢DS_{r}⊢DS_{b}⊙PS_{ba}⊙PS_{nc}⊙GFMS⊙\\ CS_{ni_{1}}⊣CS_{ni_{2}}⊣...⊣CS_{ni_{n}}⊙PS_{ps}. \end{array}$$

**Figure 22:**
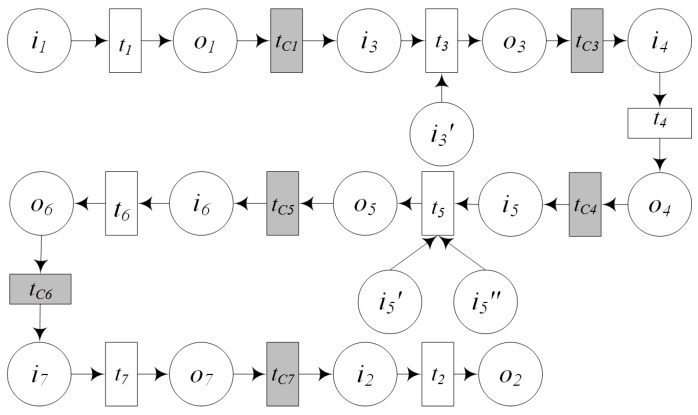
CPN model of the parking process.

Figure 22 represents the CPN model of the parking process. $i_{1}$ represent the input of GFCS; $i_{1}=\left\{S_{ei},DS_{a}\right\}$; $t_{1}$ is the internal operations of GFCS; $o_{1}$ is the composed geo-feature which represent the entrance equipment *A* of the parking lot. $i_{3}$ is the input of $PS_{ba}$ and is the geo-feature which represents the equipment *A*; $i_{3}^{\prime }$ is another input of $PS_{ba}$ and is the geo-feature set of the parking space in the parking lot; $o_{3}$ is the output of $PS_{ba}$ and is the geo-feature set of the navigating equipments $B_{r}$; $t_{3}$ is the internal operations of $PS_{ba}$ which can calculate the nearest parking space. $i_{4}$ is the input of $PS_{sr}$ and is the geo-feature which represents the nearest parking space; $o_{4}$ is the output of $PS_{sr}$ and is the geo-feature which represents the shortest route from the entrance to the nearest parking space; $t_{4}$ is the internal operations of $PS_{sr}$ which can calculate the shortest route. $i_{5}$ is the input of $PS_{ba}$ and is the geo-feature which represents the shortest route; $i_{5}^{\prime }$ is the second input of $PS_{ba}$ and is the geo-feature set of the road in the parking lot; $i_{5}^{\prime \prime }$ is the third input of $PS_{ba}$ and is the geo-feature set of the navigating equipments *B* in the parking lot; $o_{5}$ is the output of $PS_{ba}$ and is the geo-feature set which represents the navigating equipments $B_{r}$ along the shortest route; $t_{5}$ is the internal operations of $PS_{ba}$. $i_{6}$ represent the input of $PS_{nc}$; $t_{6}$ is the internal operations of $PS_{nc}$ which can calculates out the navigating commands of every navigating equipments $B_{r}$ along the shortest route; $o_{6}$ is the output geo-feature set which represents $B_{r}$ with navigating commands. $i_{7}$ is the input of GFMS, and is geo-features with control commands; $o_{7}$ is the mapped controlling services; $t_{7}$ is the internal operation of GFMS which mapping the control commands to the corresponding controlling device services; $o_{7}=\left\{CS_{ni_{1}},CS_{ni_{2}},...,CS_{ni_{n}}\right\}$. $i_{2}$ is the input of $PS_{ps}$ and is the perceived information which represents whether the free parking space is in use; $o_{2}$ is the modified parking space data; $t_{7}$ is the internal operation of $PS_{ps}$ which modifies the sate of the parking space to “in use”. $t_{C1},t_{C3},t_{C4},t_{C5},t_{C6}$ are control transitions which are used to compose the adjacent services. Then the former service can provide the input for the latter service. $t_{C7}$ is the control transition too, but it just connect the former service GFMS and the latter service $PS_{ps}$ from business logic. GFMS and $PS_{ps}$ have no data relationship.

#### 5.5.2. Example of Lake Water Pollution Control

Lake pollution is a serious problem in many countries. Generally when the water pollution is found, all kinds of physical or chemical measures would be carried out to deal with it. However, at this time the water pollution has caused a certain loss, and the pollution has a certain lag. The application of the Internet of things can make up the defects of the water pollution control. In the lake, especially in some of the sewage outfall a certain number of sensors are deployed. These sensors can sense the quality of Lake water in real time. In addition a certain number of water pollution control equipment are deployed in the lake. When the sensor detects some of contaminants in the water exceeding to a certain range, the water pollution control equipments will discharge a certain concentration of biological agents or chemical agents to the polluted water for purification. This way of using the Internet of things can deal with the lake water pollution in real time automatically.

**Figure 23:**
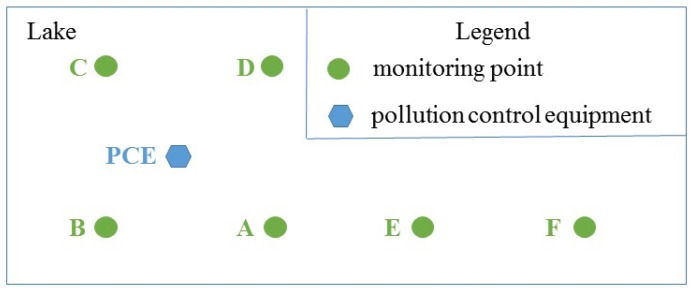
Sketch map of lake water pollution.

In the Figure 23, a large number of monitor points and pollution control equipments were deployed in the lake. Every monitor points contains sensors which can sense the the pH value, water temperature, water level, dissolved oxygen, turbidity and some other water quality indexes. All these indexes can be get by the sensing services from these sensors. So a monitor point can be regarded a geo-feature with water quality index attributes. For every water quality index, pollution control equipment can discharge the corresponding biological or chemical agent to deal with the pollution. In these pollution control equipment there are some controlling services to discharge these agents. So every pollution control equipment can be regarded a geo-feature with controlling commands.

When some sensors sense that some water quality indexes are changed to a bad range, all these sensors provide these indexes to the corresponding GIS processing service to calculate the polluted area, and calculate out the pollution control equipments in this area. Meanwhile, some GIS processing service will calculate the needed biological agents and its concentration and some other information. All these information is sent to the pollution control equipments. These pollution control equipments discharge these biological agents according the former service.

We assume there only three types of sensors which can sense the pH value, the dissolved oxygen value, the water turbidity value. Correspondingly there are three types of biological agents to deal with these three pollution indexes. Table 5 shows the needed services and Table 6 shows the attached equipments of these services.

The following equation uses Service Algebra to model the parking process.

$$\begin{array}{c} S::=S_{pH}⊢S_{do}⊢S_{wt}⊢DS_{mp}⊙GFCS⊙PS_{area}⊢DS_{pce}⊙PS_{ba}⊙PS_{bc}\\ ⊙GFMS⊙CS_{pH}⊣CS_{do}⊣CS_{wt}. \end{array}$$

**Figure 24:**
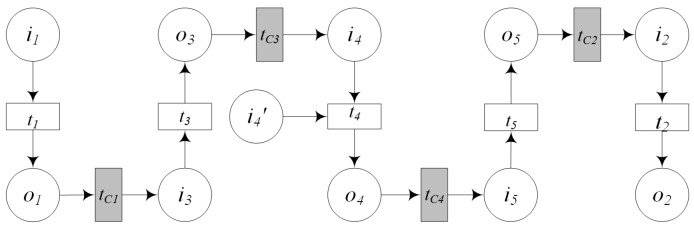
CPN model of lake water pollution control.

Figure 24 represents the CPN model of lake water pollution control process. $i_{1}$ represent the input of GFCS, $i_{1}=\left\{S_{pH},S_{do},S_{wt},DS_{mp}\right\}$; $t_{1}$ is the internal operations of GFCS which composes the former services to geo-features of the monitor point; $o_{1}$ is the composed geo-feature which represent the monitor point. $i_{3}$ is the input of $PS_{area}$ and is the geo-feature which represents the monitor points with abnormal pollution indicators; $o_{3}$ is the output of $PS_{area}$ and is the geo-feature of the polluted area; $t_{3}$ is the internal operations of $PS_{area}$ which can calculate the polluted area. $i_{4}$ is the input of $PS_{ba}$ and is the geo-feature which represents the polluted area; $i_{4}^{\prime }$ is another input of $PS_{ba}$ and is the geo-feature set of the pollution control equipments; $o_{4}$ is the output of $PS_{ba}$ and is the geo-feature set which represents the pollution control equipments in the polluted area; $t_{4}$ is the internal operations of $PS_{ba}$ which can calculate out the pollution control equipments in the polluted area. $i_{5}$ is the input of $PS_{bc}$ and is the geo-feature which represents the monitor points with abnormal pollution indicators and the pollution control equipments in the polluted area; $o_{5}$ is the output of $PS_{bc}$ and is the geo-feature set which represents the pollution control equipments with new control commands for discharging biological agents in the polluted area. $t_{5}$ is the internal operations of $PS_{bc}$. $i_{2}$ is the input of GFMS, and is the geo-feature set of pollution control equipments with control commands; $o_{2}$ is the mapped controlling services; $t_{2}$ is the internal operation of GFMS which mapping the control commands to the corresponding pollution control equipments; $o_{2}=\left\{CS_{pH},CS_{do},CS_{wt}\right\}$.

### 5.6. Discussion

The goal of this paper is to design a modeling method which can better model the interactions between the sensor device and the geo-entity in the geographic environment, can better adapt to the object-oriented processing method of GIS, and can better meet the understanding of the business staff. Under the SOA framework, we use service composition to study and model these interactions for the GIS-oriented IoT service application. The model of the service composition should reflect the interactions, should be easy to understand, should be suitable for building applications and should be evaluable.

From all above examples, we can see that the modeling method proposed by this paper can well describe the interactions between the sensor device and the geo-entity in the geographic environment under the SOA framework. These models are easy to understand and operate for the users. We can easily see which IoT services and GIS services are involved and how they are involved. Meanwhile, Some questions, for examples, whether the ways of their composition are right, what about the performances, how the data (especially the composed geo-feature) flows and changes between the sensors devices and the geo-entities in the geographic environment through IoT servies and GIS services, can be simulated well by the evaluation tool. All these aspects are very important for the next IoT services application. The following qualitatively summarizes the advantages of the modeling method proposed in this paper from the ease of use, the reusability and the evaluability:

(1) The modeling process and the established model are easy to understand and operate. The sensed object of GIS-oriented IoT service application is the geo-entity, and the basic unit of GIS spatial analysis and processing is the geo-feature. The modeling method in this paper maps the geo-entity in geographical space to the geo-feature in information space, and then maps the control commands to the geographical entity finally. In the whole business process, for both modeling staff and business application staff, the consideration is the object of geo-entity in the entire business process, and is no longer the discrete information of sensed number or string with fuzzy relations. All the sensed information related to a geo-entity is regarded as a whole object, which is more suitable for the object-oriented processing method of GIS. Therefore, this approach is very close to the business application process and is very easy to understand.
(2) The model established by this modeling method is reusable. This method regards the modeling process as a service composition process. The atomic service can be composed to a complex service, and the complex service can be composed to a more complex service. So the model or parts of the model established in this paper can be used as a service for more complex business needs. This method uses service algebra operation for service composition, so that the service composition becomes like a mathematical calculation. Services can be added or deleted easily and the order of service composition can be adjusted according to business needs at anytime.
(3) The model established by this modeling method is evaluable. CPN has been extensively studied and there has been many reliable modeling and analysis tools for CPN. CPN model can simulate all kinds of dynamic state, analyze the accessibility, fairness and boundedness properties, and is very conducive to the test and application of the model. The model established by this paper can be verified by CPN tools well.

## 6. Conclusions

With the development of IoT technology, there are more and more applications of IoT. In this paper, a new modeling method is proposed for the GIS-oriented IoT service application. The IoT service maps the state information of the geo-entity from the geographical space to the information space, and returns the operation instructions from the information space to the geo-entity. In this paper, the GIS service regards the geo-feature (the mapping of geo-entity) as the basic object of spatial analysis. Therefore, the geo-entity, the IoT service and the GIS service are closely linked to each other. According to the relationship between the IoT service and the geo-entity, the IoT service can be classified into the sensing service and controlling service. From the view of the relations between the geo-entity and the IoT service, this paper modeled the geo-feature, the two kinds of IoT services. And then this paper modeled the data service and processing service of GIS. From the view of information flow in business application, this paper discussed the relationship between them. In this paper, the process of GIS-oriented IoT service application is divided into three tasks: the sensing service composition, GIS processing service composition, controlling service mapping. This paper uses service algebra and CPN to discuss the geo-feature model, the sensing service model, the controlling service model and the composition of these three tasks. Finally, three examples are proposed to illustrate the process of modeling and the advantages of this modelling method are discussed. These examples show that this modelling method is feasible, and the purpose of this paper is achieved.

The performance of the whole sensor application system has a great challenge because of the following three reasons:

(1) The device which provides the IoT Service is resource-limited, i.e., the capacity of communication, computing, storage and so on is insufficient. In the case of insufficient resource, the quality of the service provided by the device will decline, or even become invalid.
(2) A variety of mobile terminals, such as the mobile phone, the tablet and others, connect each other through wireless Internet. Various services are running on these mobile devices, and at the same time these services can be called by other devices. This condition causes service providers and service users to change dynamically.
(3) The IoT device deployed in the field of natural environment is affected by the natural environment easily, such as the rainfall, wildlife damage, etc. The device may not be able to provide service regularly.

All these reasons cause the response time, the fault-tolerant ability and the resource consumption to become the most important factors in the performance of the IoT service. So the analysis of their performance is a very important issue and needs continuous in-depth study.

This paper proposed a modeling method for the interaction between the sensor and geographic environment. In the real application environment for the enterprise, the further analysis of the performance and establishing a practical application framework with well performance is very important. For this application framework, our ongoing work includes the following:

(1) Establishing a service calculation language. The user can describe the relationship between the IoT service and the GIS service, and describe the function of these services which reflect the interaction of the sensor and the geographic environment. The application framework can automatically generate the CPN model of the composed services according the syntax.
(2) Establishing a corresponding relationship between the service composition properties and the CPN model. The user can analyze the properties of the model. The framework can answer the user’s properties analysis request according the corresponding relationship. These properties contain completeness properties, boundedness properties, liveness properties and some others.
(3) For the real application, this framework needs the semantic model, the service discovery, the service orchestration. Meanwhile this framework must effectively deal with the performance challenge of IoT service. The CPN model should be mapped to the Business Process Execution Language (BPEL) for further application. So it is needed to develop a converting tool to convert the CPN model to BPEL.

## Figures and Tables

**Table 1:** Parameter configuration of experiment.

InitialMark	Transition	Execution Rate
(1,0,0,0,0,0,0,0,0,0)	t1	7
	$t_{2}$	8
	$t_{3}$	5
	$t_{4}$	7
	$t_{5}$	6
	$t_{C1}$	3
	$t_{C2}$	2
	$t_{C3}$	4
	$t_{C4}$	5

**Table 2:** Performance parameter.

Transition	$\bar{N}$	$\bar{\mathrm{AR}}$	Γ
$t_{1}$	0.05055863	0.31923732	0.9376223
$t_{2}$	0.12033518	0.92383003	0.56388432
$t_{3}$	0.05382902	0.76548293	0.43567223
$t_{4}$	0.07836251	0.657384723	0.53523673
$t_{5}$	0.123847364	0.45783829	0.83462718
$t_{C1}$	0.14537282	0.367382392	0.3234521
$t_{C2}$	0.083326745	0.439489283	0.43927655
$t_{C3}$	0.045736838	0.43647282	0.33654381
$t_{C4}$	0.037464836	0.32455664	0.34265467

**Table 3:** The services in the parking lot.

Type	Name	Function
IoT Sensing Service	$S_{ei}$	Sensing the license plate number by equipment *A*
	$S_{pn}$	Sensing the license plate number by equipment *B*
IoT Controlling Service	$CS_{ni}$	Sending navigation commands by equipment *B*
GIS Data Service	$DS_{a}$	Providing the entrance data of the parking lot.
	$DS_{p}$	Providing the parking space data
	$DS_{r}$	Providing the parking road data of the parking lot.
	$DS_{b}$	Providing the navigating equipments data of the parking lot.
GIS Processing Service	$PS_{ba}$	calculating the nearest parking space
	$PS_{sr}$	Calculating the shortest road
	$PS_{nc}$	Calculating the navigating commands
	$PS_{ps}$	Updating the parking space data
Assistant Service	GFCS	Sensing service composition
	GFMS	Controlling service mapping

**Table 4:** Attached equipments of the services.

Services Name	Attached Equipments
$S_{pH},S_{do},S_{wt}$	The monitor points
$CS_{pH},CS_{do},CS_{wt}$	The pollution control equipments
$DS_{mp},DS_{pce}$	GIS server
$PS_{area},PS_{ba},PS_{bc}$	GIS server
GFCS,GFMS	GIS server

**Table 5:** The services in the parking lot.

Type	Name	Function
IoT Sensing Service	$S_{pH}$	Sensing the pH value
	$S_{do}$	Sensing the dissolved oxygen value
	$S_{wt}$	Sensing the water turbidity value
IoT Controlling Service	$CS_{pH}$	Controlling the biological agents for the pH value
	$CS_{do}$	Controlling the biological agents for the dissolved oxygen value
	$CS_{wt}$	Controlling the biological agents for the water turbidity value
GIS Data Service	$DS_{mp}$	Providing the monitor points data in the lake.
	$DS_{pce}$	Providing the pollution control equipments data in the lake
GIS Processing Service	$PS_{area}$	calculating the pollution area
	$PS_{ba}$	Calculating the needed pollution control equipments
	$PS_{bc}$	Calculating the needed biological agents and its concentration
Assistant Service	GFCS	Sensing service composition
	GFMS	Controlling service mapping

**Table 6:** Attached equipments of the services.

Services Name	Attached Equipments
$S_{ei}$	The license plate number identification equipment *A* at the entrance
$S_{pn}$, $CS_{ni}$	The parking space navigation equipment *B* including many $B_{r}$
$DS_{a}$, $DS_{p}$, $DS_{r}$, $DS_{b}$	GIS server
$PS_{ba}$, $PS_{sr}$, $PS_{nc}$, $PS_{ps}$	GIS server
GFCS,GFMS	GIS server
